# Uncertainty of stochastic parametric approach to bone marrow dosimetry of ^89,90^Sr

**DOI:** 10.1016/j.heliyon.2024.e26275

**Published:** 2024-02-10

**Authors:** Elena A. Shishkina, Pavel A. Sharagin, Evgenia I. Tolstykh, Michael A. Smith, Bruce A. Napier, Marina O. Degteva

**Affiliations:** a Urals Research Center for Radiation Medicine, Chelyabinsk, Russia; b Chelyabinsk State University, Chelyabinsk, Russia; c Pacific Northwest National Laboratory, Richland, WA, USA

## Abstract

The objective of this study is to evaluate the uncertainties of the dosimetric modeling of active marrow (AM) exposure from bone-seeking ^89,90^Sr. The stochastic parametric skeletal dosimetry (SPSD) model was specifically developed to study the long-term effects resulting from chronic ^89,90^Sr exposure in populations of the radioactively contaminated territories of the Southern Urals region of the Russian Federation. The method permits the evaluation of the dose factors (*DF(AM ← TBV)* and *DF(AM ← CBV))*, which convert the radionuclide activity concentration in trabecular (*TBV*) and cortical (*CBV*) bone volumes into dose rate in the *AM*, and their uncertainties. The sources of uncertainty can be subdivided into inherent uncertainties related to the individual variability of the simulated objects and introduced uncertainties related to model simplifications. Inherent uncertainty components are the individual variability of bone chemical composition, bone density, bone micro- and macro-architecture as well as *AM* distribution within the skeleton. The introduced uncertainties may result from the stylization of bone segment geometry, assumption of uniform cortical thickness, restriction of bone geometry and the selection of the applied voxel resolution.

The inherent uncertainty depends on a number of factors of influence. Foremost, it is the result of variability of *AM* distribution within the skeleton. Another important factor is the variability of bone micro- and macro-architecture. The inherent uncertainty of skeletal-average dose factors was found to be about 40–50%. The introduced uncertainty associated with the SPSD model approach does not exceed 16% and mainly depends on the error of bone-shape stylization. The overall inherent and introduced uncertainties of *DF(AM ← TBV)* and *DF(AM ← CBV)* are below 55% and 63%, respectively. The results obtained will be incorporated into the stochastic version of the Techa River Dosimetry System (TRDS-2016MC) that provides multiple realizations of the annual doses for each cohort member to obtain both a central estimate of the individual dose and information on the dose uncertainty.

## 1 Introduction

This study is devoted to the uncertainties of bone marrow dosimetry for ^89,90^Sr with the stochastic parametric skeletal dosimetry (SPSD) modeling approach [1]. The SPSD method was specifically developed to study the long-term effects of active marrow (AM) chronic exposure to ^89,90^Sr in the populations of the radioactively contaminated territories of the Southern Urals region of the Russian Federation [2]. The contamination was the result of radioactive releases from the Mayak plutonium production facility into the Techa River during the early period of its operation (1949–1956) [3]. Ca-like isotopes were incorporated into bones resulting in irradiation of the bone marrow. Total AM doses reached 8 Gy for some individuals [4], with dominant contributors to dose being the incorporated strontium isotopes (^89,90^Sr). Previous risk analysis for the Techa River Cohort established a statistically significant dose-response association for leukemia incidence [5]. Therefore, the bone marrow dose from ^89,90^Sr is of critical importance to the leukemia risk analysis.

In general, the Techa River Dosimetry System (TRDS) determines the absorbed dose to active bone marrow, D, due to chronic radionuclide intake, $I_{r,\tau }\left(t\right)$, according to Equation (1) [6,7].

(1) $$D=\sum_{s=1}^{2}\int_{t=0}^{t_{d}}I_{r,\tau }\left(t\right)\int_{T=t}^{T_{d}}{DF}_{\tau +t}\left(AM\leftarrow s\right)R_{\tau ,s}^{\prime }\left(T\right)dTdt$$

where *t* – the time from beginning of intakes *t* = 0 to $t_{d}$ – the duration of intakes (years); *T* – the time of exposure after a single intake (years); *T*_*d*_ – the duration of exposure; $I_{r,\tau }\left(t\right)$ – individual intake function specific to residence, *r,* and age, *τ*, at the time of intake *t*; $R_{\tau ,s}^{\prime }\left(T\right)$ – specific radionuclide retention describing the fraction of radionuclide activity in the source tissue, *s,* per unit of its mass at time *T* after intake (kg^−1^), the function depends on *τ*; ${DF}_{\tau +t}\left(AM\leftarrow s\right)$ – dose factor providing age-specific (τ+t) conversion from radionuclide activity concentration in source tissues, *s,* to dose in active bone marrow, *AM,* (Gy/year per Bq/kg). The trabecular bone volume, *TBV,* and cortical bone volume, *CBV,* are assumed to be separate source tissues.

Radionuclide intakes and retention of ^90^Sr in the skeleton were derived from the available unique set of experimental data. The intake function had been reconstructed based on long-term monitoring of the ^90^Sr body-burden and tooth beta count measurements for the Techa riverside residents as well as data on the radionuclide activity concentration in drinking water and cow's milk, which were summarized and analyzed in Ref. [8]. Additionally, ^90^Sr intake for breast-fed infants was reconstructed [9,10]. The age-dependent model for strontium retention in human bone was evaluated in Ref. [11]. Details of the study of strontium metabolism in humans have been described in Ref. [12]. The biokinetic model for strontium, which takes into account the age and gender differences in bone mineral metabolism, was described in Ref. [7].

Until now dose factors for ^89,90^Sr used in TRDS were taken from published data [13,14]. They are the result of an estimation of the continuous deposition of beta particle kinetic energy while it travels across both bone trabeculae and marrow. The measurements of the frequency distributions of linear path lengths (chord-length distributions) in trabecular bone served as the basis of the one-dimensional modeling of electron transport. This technique is considered obsolete in modern dosimetric modeling. There are more advanced tools allowing 3D Monte Carlo simulation of radiation transport taking into account the angular deflection and fluctuations of energy loss of charged particles [15]. Reevaluation of *DFs* is one of the key issues resolved with the SPSD approach. Dose factors are assessed through radiation transport simulation using computational phantoms. The main concept of the SPSD approach is to create parametric computational phantoms of hematopoietic bone segments for calculation of bone-specific dose factors, which can be averaged by weights proportional to *AM* fraction. Parameters of spongiosa microarchitecture, cortical bone thickness as well as bone linear dimensions were evaluated using a vast amount of morphometric data. Literature-derived parameters are used for the random generation of the rod-like trabecular structure of spongiosa inscribed into stylized bone shapes covered by a uniform layer of cortical bone [16,17].

Note that the methodology most commonly used for internal dosimetry in contemporary literature [[18], [19], [20]] is based on a formalized approach established by The Medical Internal Radiation Dose (MIRD) Committee [21,22]. According to MIRD, the dose to a target organ (D) from a source region (s) is the product of the cumulated radionuclide activity (i.e., the number of decays) in the source region (${\tilde{A}}_{s}$) and the absorbed dose (*S)*. The absorbed dose to *AM* per unit cumulated activity in the source region (*s)* can be written as Eqn. (2).

(2) $$D\left(AM\leftarrow s\right)={\tilde{A}}_{s}\times S\left(AM\leftarrow s\right)$$

The main purpose of medical dosimetry is to obtain a patient-specific dose estimate for a given bone or bone group. On the contrary, the dosimetry for analytical epidemiological studies requires an unbiased population-specific and skeletal-average factor. The TRDS method uses a more intensive parameter, namely, dose factor DF(AM←s) (as described in Eqn. (1)).

Contemporary epidemiological studies are based on a dosimetry [23] that provides both point estimates and uncertainties of individual doses. The estimation of uncertainty of bone marrow dosimetry for bone-seeking beta emitters is an issue of high priority and it is a challenge. In this sense, the parametric approach to creating phantoms and understanding the limits of individual variability of anthropometric parameters [24] allows for the generation of sets of random models reflecting the individual variability of bone micro and macro dimensions. Voxel phantoms of bone segments were generated in a specially designed computer program ‘Trabecula’ [25]. Individual variability of segment-specific phantom parameters results in uncertainty of DF(AM←s); in other words, the natural individual variability of bone morphology is the source of inherent uncertainty of a population-average dose factor and this uncertainty could not be reduced. Besides natural variability, additional sources of uncertainty result from simplification of bone shape, assumption of uniformity of cortical thickness and a number of other model approximations.

The purpose of this study was to evaluate the uncertainties (both, inherent and those introduced by the SPSD modeling approach) of bone marrow dose factors for ^89.90^Sr incorporated in the trabecular and cortical bone tissues. To achieve this purpose, all significant contributors to uncertainty should be identified and evaluated.

## 2 General SPSD approaches

### 2.1 SPSD computational phantoms

The mean electron energy of ^90^Sr+^90^Y decay is 564.6 MeV. This is similar to that of ^89^Sr decay (584.9 MeV). According to Ref. [26], the mean free path length of electrons (continuous slowing-down approximation) of ^90^Sr+^90^Y decay (as well as of ^89^Sr decay) in spongiosa can be expressed by Eqn. (3) and could take on a value from 0.15 to 0.2 cm.

(3) $$\lambda \left(\bar{E}\right)=0.112+0.099\times e^{-1.526\frac{BV}{TV}},\left(cm\right)$$

where BV/TV is the bone volume fraction of spongiosa.

**Fig. 1:**
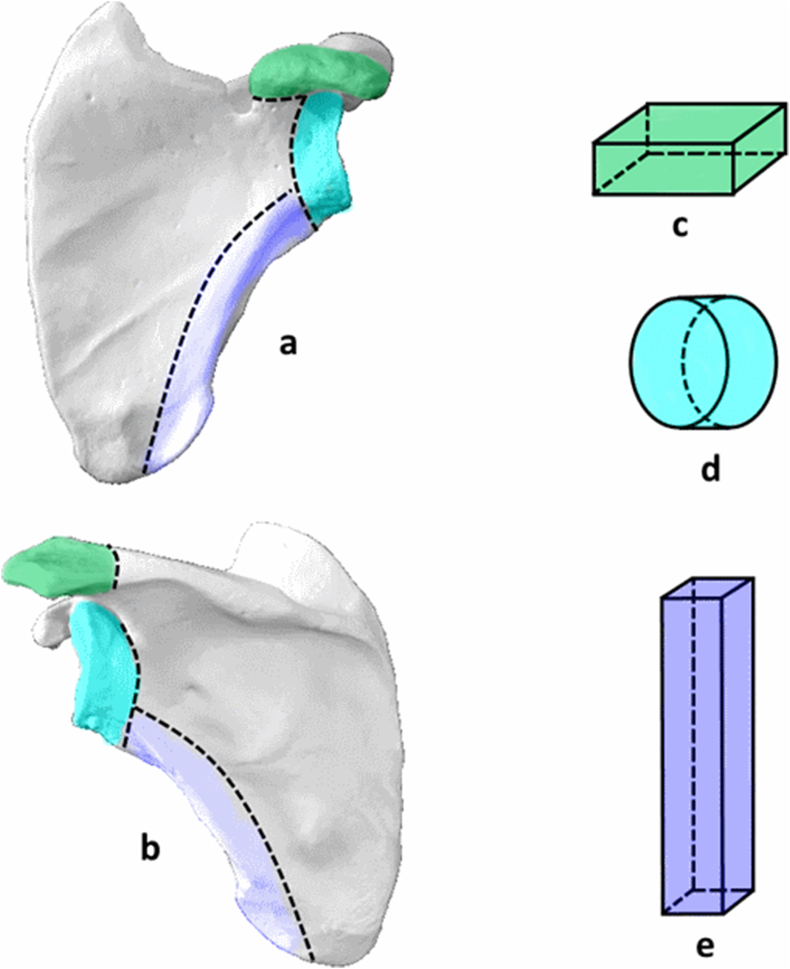
Example of segmentation and stylization of scapula. Bone segments with active hematopoiesis in the adult scapula are colored; a – view of the scapula from the front, b – view of the scapula from behind, c, d, e − phantoms of segments acromion, glenoid and lateral margin, respectively. The color of the phantoms (c, d, e) corresponds to the color of the bone segments (a, b). (For interpretation of the references to color in this figure legend, the reader is referred to the Web version of this article.)

The maximum electron pathlength [26], λ(Q), is approximately equal to $5\times \lambda \left(\bar{E}\right)$ and does not exceed 1.1 cm. This makes it possible to avoid modeling the entire skeleton and to focus on the radiation transport within the separate bones or bone segments. Therefore, only hematopoietic sites were considered. Each of the complex-shaped bone sites under consideration [1] is subdivided into a set of simple-shaped segments with mostly homogeneous and isotropic microstructure [27]. Such a segment could be described by simple geometry (Table 1). The shapes selected for segment-specific phantoms depends on the age of the skeleton simulated and were most often well described by a box, cylinder or deformed cylinder (Table 1). As an example, Fig. 1(a–e) illustrates the bone segmentation and stylization used to model the scapula.

**Table 1:** Shapes of stylized phantoms and fraction of segment-specific phantoms of different shape among all simulated phantoms (of different sex and age).

Shape	Comment	Fraction, % mean [range]
Box	Rectangular parallelepiped	47 [32–61]
Cylinder	Can be circular- or elliptic based	30 [[28], [29], [30], [31], [32]]
Deformed cylinder	A figure of irregular shape with two parallel elliptical bases and ruled lateral surface. The planes on which parallel axes lie are perpendicular to each other and the planes of the bases. A truncated cone is a particular case of deformed cylinder.	21 [[11], [12], [13], [14], [15], [16], [17], [18], [19], [20], [21], [22], [23], [24], [25], [26], [27], [33]]
Triangular pyramid	Pyramids are only used to describe a segment of pelvic *ischium acetabulum* and a segment of *sacral ala*	2% [0–3]
Ellipsoid	Typical of talus and calcaneus of both hand and foot; considered as hematopoietic for newborn only	<0.6 [0–3]
Tube	A figure bounded by the two nested cylinders; typical of pelvic acetabulum only.	1 [0–2]

Stylized phantoms include spongiosa covered by a cortical layer [16]. The rod-like trabecular microstructure of the stochastic spongiosa model is described with the following parameters.

- *Tb*.*Th*, *σ*_*Tb*.*Th*_ – mean trabecular thickness and its intra-specimen variability;
- *Tb*.*Sp*, *σ*_*Tb*.*Sp*_ – mean trabecular separation and its intra-specimen variability
- *BV/TV, min-max* – bone volume fraction of spongiosa and the range of possible values

The cortical layer does not cover all the surfaces of the phantom, but only those where it is present in the simulated bones. The cortex is represented by homogeneous and isotropic bone substance located between two surfaces. One of them is the outer boundary of the stylized phantom, and the other is located inside the phantom. The distance between them is equal to the mean cortical thickness (*Ct.Th*). The outer boundaries of phantoms depend on segment-specific linear dimensions. Each of the parameters is segment-specific and derived from literature [24,27].

It is clear that the average morphometric parameters of phantoms are uncertain in terms of individual variability (inherent uncertainty). Besides, the geometric description may introduce some errors. In addition to the geometric description, phantoms are characterized by the chemical composition and density of the media where electron transport will be simulated. These parameters also contribute to uncertainties. The phantoms are generated as voxel representations that can also be a source of introduced uncertainty.

2.2. Conversion of radionuclide activity to dose.

Energy deposition in bone marrow is calculated by simulating radiation transport for bone segment, *i,* taken from site, *j,* with segment-specific computational phantoms. Segment-specific dose factors, ${DF}_{i,j}\left(AM\leftarrow s\right)$, are calculated according to Eqn. (4) assuming a uniform distribution of *AM* within the marrow of the segments.

(4) $${DF}_{i,j}\left(AM\leftarrow s\right)=E_{i,j}\left(BM\leftarrow s\right)\times \frac{m_{s}}{m_{BM}}$$

where $E_{i,j}\left(BM\leftarrow s\right)$ is the mean energy absorbed in bone marrow (*BM*) per decay of radionuclides incorporated in source tissue *s* (trabecular or cortical bone); $m_{s}$ – mass of source tissue; $m_{BM}$ – mass of bone marrow. For ^90^Sr, energy deposition due to electrons of combined ^90^Sr+^90^Y spectrum per decay of parent radionuclide was considered.

${DF}_{i,j}\left(AM\leftarrow s\right)$ is an intensive variable that is practically insensitive to tissue mass variation. Source and target masses are dependent values and the use of their ratio minimizes the effect of an error of tissue mass estimation. This is especially significant for spongiosa, where trabecular bone and bone marrow are structurally interrelated.

Dose factors calculated for different segments, *j,* of one site, *i* (for example segments of scapula in Fig. 1), are averaged with weights equal to normalized segment-specific bone marrow masses (Eqns. (5), (6))) considered in the phantoms.

(5) $${DF}_{i}\left(AM\leftarrow s\right)=\sum_{j=1}^{n_{i}}{w_{i,j}DF}_{i,j}\left(AM\leftarrow s\right)$$

and

(6) $$w_{i,j}=\frac{{m_{BM}}_{i,j}}{\sum_{j=1}^{n_{i}}{m_{BM}}_{i,j}}$$

where *n*_*i*_-number of segments of site *i*; ${m_{BM}}_{i,j}$ – mass of bone marrow in segment *j* of site *i*; $w_{i,j}$ - weights.

Skeletal-average dose factors, DF(AM←s), were calculated as weighted average of site-specific values (${DF}_{i}\left(AM\leftarrow s\right)$). Weights (*w*_*i*_) were assigned proportionally according to literature-derived site-specific fractions of *AM* [33,34] (Eqn. (7)). The details were presented in Ref. [1].

(7) $$DF\left(AM\leftarrow s\right)=\sum_{i=1}^{n}\frac{w_{i}{DF}_{i}\left(AM\leftarrow s\right)}{\sum_{1}^{n}w_{i}}=\sum_{i=1}^{n}W_{i}{DF}_{i}\left(AM\leftarrow s\right)$$

where n – is the number of sites under consideration; *W*_*i*_*=* $\frac{w_{i}}{\sum_{1}^{n}w_{i}}$ is normalized weight. Examples of dose factor calculations for adults are published in Refs. [4,17].

Site-specific *AM* fraction is an additional geometry-independent parameter of a skeletal computational phantom. The overall uncertainty of these parameters includes the inherent variability of the *AM* distribution and may have an introduced error (a possible non-excluded systematic error) of *w*_*i*_ estimate.

2.3. Structure of DF(AM←s) uncertainty

The sources of uncertainties can be subdivided into inherent and introduced. The overall uncertainty of DF(AM←s) is estimated in several stages. At first, the relative standard uncertainties of segment-specific ${DF}_{i,j}\left(AM\leftarrow s\right)$ should be evaluated. Inherent sources of uncertainties are as follows: variability of bone micro and macro dimensions, variability of tissue density and chemical composition. Monte Carlo simulation of radiation transport may be sensitive to geometry simplification including shape stylization and uniform thickness of cortical layer, restricted volume of spongiosa due to segmentation and voxel resolution of computational phantoms.

The skeletal average dose factors have an additional source of uncertainty, namely, the uncertainty of weights (*w*_*i*_) assigned. Standard uncertainties (σ) of *DF=* $\sum_{i=1}^{n}W_{i}{DF}_{i}\left(AM\leftarrow s\right)$ were estimated conservatively using uncertainty propagation considering the independence of the sources of uncertainties. According to Ref. [35], linear dimensions and masses of various skeletal bones are correlated. Therefore, the ratios of bone tissues and bone marrow (source and target tissues) of different sites should also correlate, as well as the corresponding dose factors (see Eqn. (4)). The mean correlation coefficient is about 0.5 [35]. Standard uncertainty can be formulated as follows:

$$\sigma^{2}=\sum_{i=1}^{n}\left[{\left(\frac{\partial \left(W_{i}{DF}_{i}\left(AM\leftarrow s\right)\right)}{\partial W_{i}}\right)}^{2}{\sigma_{W_{i}}}^{2}+{\left(\frac{\partial \left(W_{i}{DF}_{i}\left(AM\leftarrow s\right)\right)}{\partial {\bar{DF}}_{i}\left(AM\leftarrow s\right)}\right)}^{2}{\sigma_{{\bar{DF}}_{i}}}^{2}\right]+\sum_{i}^{n}\sum_{l\left(l\neq i\right)}^{n}{0.5\sigma }_{i}\sigma_{l}=$$

$$\sum_{i=1}^{n}\left[{{DF}_{i}\left(ККМ\leftarrow s\right)}^{2}{w_{i}}^{2}\left(\delta_{w_{i}}^{2}+\delta_{{DF}_{i}}^{2}\right)\right]+\sum_{k}^{n}\sum_{l\left(l\neq k\right)}^{n}{0.5\sigma }_{i}\sigma_{l}$$

where $\delta_{W_{i}}^{2}\;and\;\delta_{{\bar{DF}}_{i}}^{2}$ are relative standard uncertainties of weight factors and site-specific dose factors.

The relative standard uncertainty of DF(AM←s) can be expressed conservatively by Eqn. (8):

(8) $$\delta =\frac{\sqrt{\sum_{i=1}^{n}\left[{{DF}_{i}\left(ККМ\leftarrow s\right)}^{2}{w_{i}}^{2}\left(\delta_{w_{i}}^{2}+\delta_{{DF}_{i}}^{2}\right)\right]+\sum_{k}^{n}\sum_{l\left(l\neq k\right)}^{n}{0.5\sigma }_{i}\sigma_{l}}}{\sum_{i=1}^{n}\left({DF}_{i}\left(AM\leftarrow s\right)W_{i}\right)}$$

where $\delta_{w_{i}}$ is the site-specific relative uncertainty of weighting factors (*w*_*i*_*)* reflecting the uncertainty of both variability of *AM* distribution among the hematopoietic sites and introduced uncertainty. The values of $\delta_{{\bar{DF}}_{i}}$ correspond to relative uncertainties of site-specific ${\bar{DF}}_{i}\left(AM\leftarrow s\right)$ and comprise all other sources of uncertainties (both introduced and inherent). The total number of bone sites is designated as *n*.

## 3 Materials and methods

### 3.1 Computations

Phantoms were generated using the “Trabecula” software [25] with a user-provided resolution. Bone and bone marrow chemical compositions as well as tissue densities were taken according to Ref. [36] as basic parameters for most of the computations (Table 2). Electron-photon transport was calculated with MCNP6.2 using a pulse-height tally to calculate the energy deposition in target tissue. The emission spectra of ^89,90^Sr and ^90^Y were taken from the Java-based nuclear information system (JANIS 4.1) which is an open access resource available at https://www.oecd-nea.org/jcms/pl_39910/janis [37]. The decays of ^90^Sr and ^90^Y were modeled with an equal probability to simulate secular equilibrium. Energy deposition was calculated per decay of parent radionuclide. The number of source particle histories was at least 4,000,000; statistical error <1%.

**Table 2:** Element mass fractions of simulated media: basic compositions are according to Ref. [36]. Alternative bone composition is the same as for tooth dentin [38]; alternative bone marrow composition is similar to water.

Atomic No.	Element	Basic composition		Alternative composition	
		Bone	Bone marrow	Bone	Bone marrow
1	H	0.035	0.105	0.012	0.11
6	C	0.16	0.414	0.028	
7	N	0.042	0.034	–	
8	O	0.445	0.439	0.44	0.89
9	F	–	–	0.0096	
11	Na	0.003	0.001	0.007	
12	Mg	0.002	0.002	0.011	
14	Si	–	–	0.000007	
15	P	0.095	0.002	0.16	
15	S	0.003	0.002	–	
17	Cl	–	–	0.0074	
19	K	–	–	0.0007	
20	Ca	0.215	–	0.331	
26	Fe	–	–	0.0000067	
30	Zn	–	–	0.00017	
Density, g cm^−3^		1.9	0.98	1.9	0.98

### 3.2 Effect of individual variability of model parameters

#### 3.2.1 Individual variability of chemical composition

A simple box model was used with mean morphometric parameters as follows: linear dimensions are 1.1 × 0.6 × 3 cm; *Ct.Th* = 0.08 cm; *Tb.Th* = 90 μm, *Tb.Sp* = 900 μm and *BV/TV* = 0.09. Two computations of DF(AM←s) were performed with basic and alternative chemical composition and fixed tissue density (Table 2). Alternative bone composition is taken the same as for tooth dentin [38]; alternative bone marrow composition is assumed to be the same as for water.

#### 3.2.2 Variability of bone density

Effect of bone density variability was considered for "large" (linear dimensions are > $2\times \lambda \left(\bar{E}\right)$) and “small” phantoms separately. For “large” phantoms, the dimensions and shape of a segment do not play an important role in energy loss because 95% of the radiated energy is completely absorbed inside the volume of the phantom. The effect of medium density on DF(AM←s) was estimated based on a theoretical consideration assuming the individual variability of bone density is 3% for people of the same age [[28], [29], [30]].

For “small” phantoms, the effect of bone density variation was estimated conservatively by comparing the Monte Carlo calculations for 6 different models with bone density typical of newborn (1.65 g cm^−3^) and adults (1.9 g cm^−3^). Descriptions of the phantoms are shown in Table 3.

**Table 3:** Description of ‘small’ phantoms. *TBV*, *BMV* and *CBV* are trabecular bones, bone marrow and cortical bone volumes, respectively.

N	Shape	Linear dimensions^a^, cm	$\rho_{bone}$, g cm^−3^	*Tb.Th*, cm	*Tb.Sp*, cm	*BV/TV*	*Ct.Th*, см	*TBV*, cm^3^	*BMV*, cm^3^	*CBV*, cm^3^
1	elliptic cylinder	0.38 × 0.38 × 8.9	1.65;1.9	0.012	0.0248	0.22	0.024	0.017	0.060	0.024
2	ellipsoid	0.39 × 0.39 × 0.61	1.65;1.9	0.012	0.0248	0.22	0.024	0.073	0.256	0.060
3	elliptic cylinder	0.6 × 0.6 × 3	1.65;1.9	0.01	0.036	0.28	0.13	0.076	0.196	0.577
4	deformed cylinder	1.72 × 0.6 × 0.6 × 0.6 × 1.34	1.65;1.9	0.01	0.036	0.28	0.032	0.171	0.445	0.115
5	elliptic cylinder	1.12 × 1.12 × 0.3	1.65;1.9	0.016	0.0538	0.22	0.23	0.226	0.804	1.929
6	elliptic cylinder	0.91 × 0.91 × 3	1.66;1.9	0.0174	0.058	0.22	0.16	0.184	0.640	1.130

a a × b × H – for elliptic cylinders; a × b × c × d × H for deformed cylinders and l × m × n for ellipsoid, where a and c – major axis of elliptic bases; b and d – minor axes of elliptic bases; H – height; l, m and n are ellipsoid axes.

#### 3.2.3 Variability of bone segment micro and macro dimensions

The SPSD approach simulates the individual variability of macro and microarchitecture of bone segments using the random generator of computational phantoms ‘Trabecula’ [25]. Basic segment phantoms of adult man [27] were supplemented with a set of additional random models to be used in Monte Carlo simulation of electron transport. The selection of micro and macro parameter values was performed with lognormal and normal approaches respectively, using a 90% confidence interval (i.e., 10% of the values associated with the tails of the distributions were considered as outliers). Macro parameters positively correlated; micro parameters negatively correlated (*Tb.Sp* increases with *Tb.Th* decrease). The inverse transform method was used for simulation of correlated distributions. Each of the resulting models is verified to ensure the calculated *BV/TV* ratio remains within the range of possible values.

Uncertainties of segment-specific dose factors (${\delta_{var}}_{i,j})$ were estimated as the relative root mean square deviation of dose factors of *n* randomly generated models from $\bar{{DF}_{i.j}}$ calculated with population-mean model parameter values (Eqn. (9)).

(9) $${\delta_{var}}_{i,j}=\frac{{RMSD}_{i,j}}{\bar{{DF}_{i,j}}}=\frac{1}{\bar{{DF}_{i,j}}}\sqrt{\frac{\sum_{k=1}^{n}{\left({\bar{{DF}_{i,j}}-DF}_{i,j,k}\right)}^{2}}{n}}$$

where ${DF}_{i,j,k}$ – dose factor estimated with model *k* from *n* randomly generated segment-specific phantoms simulating the individual variability.

At first, we estimated the minimum suitable number of randomly generated models, *n*. For this purpose, a simple box model was used as a basic phantom with mean parameter values as follows: linear dimensions 1.1 × 0.6 × 3 cm; *Ct.Th* = 0.08 cm; *Tb.Th* = 0.009 cm; *Tb.Sp* = 0.09 cm and *BV/TV* = 0.09. Thirty-seven supplementary phantoms were generated automatically using ‘Trabecula’ [25] assuming parameter variability to be similar to real bones. *Tb.Th* and *Tb.Sp* variation was fixed within 4%. As a result, the *BV/TV* varied within 16%. Linear dimensions varied independently within 16–17%. Cortical thickness varied within 42%. Assuming trabecular bone as the main dose contributor for bone marrow, the population average and 37 variants of DF(AM←TBV) were evaluated. Because multiple DF realizations are used to estimate its variability, the normalized root of squared deviation of randomized *DFs* from the population average was tested as a function of sample size (*n)*. The minimum acceptable sample size corresponds to the value of *n* for which the variance is insensitive to sample size (can be assumed as a constant). A quantity called the relative variance of variance (VOV) is another useful indicator that has been used [31,32]. The VOV value should be less than 0.15. The dependences of DF(AM←TBV) variance as well as VOV on sample size *n* were calculated using the bootstrap method (i.e., random sampling of *DF* dataset with replacement).

Next, individual variability was simulated by perturbing the input parameters of the model *n* times for each bone segment of the adult male. As shown in Ref. [17], the adult skeleton consists of 12 hematopoietic sites, which can be subdivided into 47 segments (a total of 47 basic segment phantoms). Table 4 illustrates the number of bone segments considered in different hematopoietic sites of adult male. The shape and parametric description of each segment, as well as the parameter variabilities, are shown in Supplemental material S1.

**Table 4:** Number of basic segment phantoms generated for 12 hematopoietic sites of adult male skeletal model.

#	Site	number of segments
1	Skull	1
2	Cervical vertebra	3
3	Thoracic vertebra	5
4	Lumbar vertebra	5
5	Sternum	2
6	Pelvic bones	8
7	Sacrum	10
8	Proximal femora	2
9	Proximal humeri	1
10	Scapulae	3
11	Clavicles	3
12	Ribs	4
Total skeleton		47

#### 3.2.4 Variability of AM distribution among hematopoietic sites

Skeletal-average dose factors are weighted by site-specific *AM* fraction (Eqn. (8)). *In-vivo* measurements of *AM* distribution within adult skeletons (and corresponding individual variability) were performed by Campbell et al. [34] using positron-emission tomography with a fluorothymidine label. These data were used in calculations of *DF* for adults [2,17]. Unfortunately, no similar data were available for children. A quantitative description of *AM* distribution depending on age was published by Cristy [33] based on a collection of histologic data on age-specific marrow cellularity for specific bones, together with information on relative volumes of body regions at different ages. However, no data on individual variability of *AM* distribution were reported. The variability typical of adults was propagated to other ages with the addition of non-excluded bias estimated by comparing the mean values from Campbell et al. [34] and Cristy [33] for adults.

### 3.3 Introduced uncertainty

#### 3.3.1 Effect of voxel resolution

A bone model of cylindrical shape was generated and voxelized with different resolutions (from 75 to 300 μm; step 25 μm). Model parameters were as follows: 4.7 × 3.5 × 2.7 cm (*a×b×H*, where *a* – the major axis of elliptic base of cylinder; *b* – the minor axis and *H* – the height); *Ct.Th* = 0.19 cm; *Tb.Th* = 200 ± 20 μm; *Tb.Sp* = 780 ± 40 μm; *BV/TV* = 0.17. The sensitivity of DF(AM←s) estimates to voxel resolution was tested.

#### 3.3.2 Stylization of bone geometry

Ideally, uncertainty due to simplification of bone shapes could be derived from a comparison of the data obtained using “true-shaped” and stylized computational phantoms. However, no “true-shaped” phantoms are available in the framework of the current study. Therefore, the uncertainties were roughly estimated *ad maximum* combining sets of segment-specific computational phantoms of different shapes and sizes. However, boxes circumscribed around the models should have similar linear dimensions.

The segment-specific phantoms were obtained in the process of constructing a skeletal phantom of an adult male (Table 4). Fifty six percent of bone segments are boxes, 27% are cylinders with an elliptical base, and 9% are deformed cylinders. The remaining segments were hemispheres or triangular pyramids. Each of the segments was modeled 13 times: one basic and 12 supplementary phantoms. The supplementary phantoms were generated based on perturbations of the parameters of the segment-specific models. A few of the supplementary segments were generated with erroneous (i.e., nonsensical) dimensions and were not included in the final version of the skeletal phantom. However, such models turned out to be useful for solving this particular problem. We consider different geometric objects of similar size to evaluate the effect of shape and size variation on *DF*. Therefore, the anatomical correspondence of a particular model is not important. In total, 676 phantoms were used in the analysis.

#### 3.3.3 The assumption of cortical thickness uniformity

Our calculations assumed a homogeneous cortical layer of uniform thickness. This approach was tested with a set of cubic models with the following edge lengths (*L*): 0.7, 1, 1.2 and 2.1 cm. As the first step, each of the cubic models was covered by a uniform cortical layer of a given thickness, $\bar{Ct.Th}$, (basic model). Then, each face of the cubic models was covered by a cortical layer of a different thickness (12 supplementary models). The surface-specific *Ct.Th*s were selected randomly and normalized in such a way that the mean *Ct.Th* of each of the supplementary models was equal to the $\bar{Ct.Th}$ of the basic model.

Two values of $\bar{Ct.Th}$ were applied for each of the cubes, namely, 0.035 cm and 0.084 cm (typical dimensions). Spongiosa microstructure parameters were all the same: *Tb.Th* = 100 μm; *Tb.Sp* = 600 μm; *BV/TV* = 0.15.

#### 3.3.4 Restriction of bone geometry

Restricted spongiosa radiation transport may result in an underestimation of the energy deposition in *AM* due to beta emission in adjacent *TBV* [39]. Volchkova et al. [40] described the adjustment coefficient and corresponding uncertainty that is used in the current study.

## 4 Results

### 4.1 Effect of individual variability of model parameters

#### 4.1.1 Chemical composition

The variability in bone chemical composition typical for tooth dentin results in *DF* differences of not more than 3.3% for both DF(AM←TBV) and DF(AM←CBV) calculated for ^90^Sr+^90^Y and ^89^Sr spectra [41]. Therefore, the uncertainty of *DF*s due to individual variability of bone chemical composition was conservatively assigned as 4%.

#### 4.1.2 Bone density

Density as a factor influencing the dose coefficient should be considered for both energy deposition $E_{i,j}\left(BM\leftarrow s\right)$ and the mass ratio of the source and target tissues (Eqn. (4)). Changes in bone density in the range of 1.65–1.9 g cm^−3^ at a given *BV/TV* do not lead to a significant change in the mean density of spongiosa (<4%) due to the large marrow fraction. The mean pathlength of ^90^Sr+^90^Y electrons as well as for ^89^Sr electrons in spongiosa (0.16–0.22 cm) greatly exceeds *Tb.Th* (0.0075–0.029 cm). For “large” phantoms the mean *BM* energy deposition per decay in the trabecular bone volume ($E_{i,j}\left(BM\leftarrow TBV\right)$) weakly depends on bone density (at least at a given range of *Tb.Th*). Therefore, the bone density effect on energy deposition in *BM* can be ignored. However, the bone to *BM* mass ratio varies proportionally to bone density variation. Variability of bone density within 3% results in the same uncertainty of *DF*_*i,j*_*(AM ← TBV)* in “large” phantoms. Variability of bone density within 3% should effect *DF*_*i,j*_*(AM ← CBV)* in “large” phantoms even less. Typically, *Ct.Th* is comparable to the mean pathlength of ^90^Sr+^90^Y and ^89^Sr electrons in bone tissue. Increases in bone density lead to a decrease of energy deposition in bone marrow. At the same time, the source to target tissue mass ratio will increase proportionally to an increase in density. In other words, *DF*_*i,j*_*(AM ← CBV)* comprises two multipliers that change in the opposite direction depending on bone density. As a result, for “large” segments the uncertainty of *DF*_*i,j*_*(AM ← CBV)* due to density variability can be neglected. The calculations and justifications are described in Ref. [41].

Energy deposition in *BM* of “small” segments can be more sensitive to density variance for both *E*_*i,j*_*(AM ← TBV)* and *E*_*i,j*_*(AM ← CBV)* due to some energy loss, which also depends on segment size, shape and *BV/TV* value. Table 5 shows the comparison of *DF*s of ^90^Sr calculated for “small” bone segment models with minimum and maximum age-specific bone densities.

**Table 5:** Comparison of *DF*s of^90^Sr calculated for “small” bone segment models with minimum and maximum age-specific bone densities.

N	*DF*_*i,j*_*(AM ← TBV)*, × 10^−11^ (Gy s^−1^)/(Bq g^−1^)		Difference, %	*DF*_*i,j*_*(AM ← CBV)*, × 10^−11^ (Gy s^−1^)/(Bq g^−1^)		Difference, %
	*ρ* = 1.65 g cm^−3^	*ρ* = 1.9 g cm^−3^		*ρ* = 1.65 g cm^−3^	*ρ* = 1.9 g cm^−3^	
1	1.69	1.88	11	2.58	2.92	13
2	1.31	1.45	10	3.66	4.1	12
3	6.21	6.53	5	3.63	4.07	12
4	1.3	1.41	8	4.69	5.22	11
5	4.92	4.91	0	5.3	4.22	20
6	4.8	4.93	3	3.57	3.96	11
Mean difference (range)			6 (0–11)			13 (11–20)

As shown in Table 5, the average difference between the estimates for *DF*_*i,j*_*(AM ← TBV) using* the minimum and maximum bone density is equal to 6%. In contrast, *DF*_*i,j*_*(AM ← CBV)* appears to be more sensitive to bone density, with a mean difference between the two estimates of 13%. The same mean differences were found for ^89^Sr. These values can be taken as the uncertainties of segment-specific *DF*s due to individual variability of bone density for “small” segments.

#### 4.1.3 Bone segment micro and macro dimensions

**Fig. 2:**
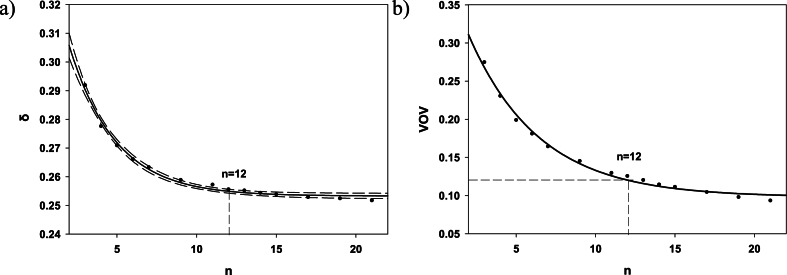
Results of multiple realizations of *DF*_*i,j*_*(AM ← TBV)* for ^90^Sr: a) the normalized RMSD, δ, as a function of sample size, *n*; the solid line is an exponential smoothing, the dashed lines border the 90% confidence interval; b) the relative variance of variance (VOV); the solid line is an exponential smoothing; the dots are results of numerical experiment.

*DF*_*i,j*_*(AM ← TBV)* equal to 0.97 × 10^−11^ Gy/s per Bq/g was calculated for ^90^Sr with a simple box model with mean parameter values as follows: linear dimensions 1.1 × 0.6 × 3 cm; *Ct.Th* = 0.08 cm; *Tb.Th* = 0.009 cm; *Tb.Sp* = 0.09 cm and *BV/TV* = 0.09. This value was considered as a “true” value. Multiple realizations of individual segment-specific *DFs* considering the individual variability of micro and macro dimensions result in the range of values from 0.69 to 1.38 ( × 10^−11^ Gy/s per Bq/g). In other words, a two-fold difference in dose coefficients may be due to variation in model parameter values. Fig. 2a illustrates the dependence of the normalized root mean square deviation (RMSD) of randomized *DFs* from the “true” value (δ) on sample size (*n)*. When *n* reaches the value 12, δ becomes independent of sample size (the 90% confidence interval of data smoothing at *n* = 12 overlaps with those at *n* > 20) and can be assumed as constant δ ~ 0.25. VOV at *n* = 12 is equal to 0.12 (Fig. 2b). This means the error of δ estimate at *n* = 12 is ∼12% and does not exceed 15%. In other words, the evaluation of the effect of individual variability of segment-specific micro and macro dimensions requires at list 12 geometry variants in addition to a population-average one.

Further, by drawing 12 additional models for each of the 47 bone segments of an adult male, it was found that ${\delta_{var}}_{i,j}$ ranged from 7% to 62% depending on the variability of input parameters of the segments (Supplemental material S1). Table 6 illustrates the segment specific individual variability of *DF*_*i,j*_*(AM ← TBV)* and *DF*_*i,j*_*(AM ← CBV).*

**Table 6:** Distribution of segment-specific *DFs* calculated for^90^Sr and corresponding ${\delta_{var}}_{i,j}$ in the computational phantom of adult male.

	DF(AM←TBV), × 10^−11^ Gy/s per Bq/g	${\delta_{var}}_{i,j}\left(AM\leftarrow TBV\right)$	DF(AM←CBV), × 10^−11^ Gy/s per Bq/g	${\delta_{var}}_{i,j}\left(AM\leftarrow CBV\right)$
mean	3.78	0.22	3.01	0.20
range	1.64–9.64	0.05–0.62	0.38–10	0.05–0.58
5%	2.11	0.12	0.65	0.07
25%	2.84	0.17	1.32	0.13
median	3.59	0.21	2.51	0.18
75%	4.19	0.24	3.64	0.25
95%	6.65	0.43	8.09	0.38

Dose factors calculated for ^89^Sr were about 2 times lower than that for ^90^Sr. Although the mean energy of ^89^Sr decay is similar to the mean energy of the combined ^90^Sr+^90^Y spectrum in secular equilibrium, the mean absorbed energy in *AM* due to ^90^Sr+^90^Y is normalized per Bq of only ^90^Sr (decay of parent radionuclide). The relative uncertainty, ${\delta_{var}}_{i,j}$ calculated for ^89^Sr is practically the same as for ^90^Sr. Therefore, subsequent descriptions of the uncertainties will utilize examples of ^90^Sr.

The shape of the *DF* distribution cannot be estimated with certainty based on 12 trials only. It is preferable to combine all of the available data on dose factors calculated with basic and supplementary segment-specific phantoms. However, segment-specific dose factors may differ by a factor of 6 for DF(AM←TBV) and even by a factor of 26 for DF(AM←CBV) (Table 6). Therefore, the pooling of segment-specific dose factors was performed after a linear transformation (Eqn. (10)).

(10) $${\tilde{DF}}_{i,j,k}=\frac{{{\bar{DF}}_{i,j}-DF}_{i,j,k}}{{\sigma_{var}}_{i,j}}$$

where ${\tilde{DF}}_{i,j,k}$ – linearly transformed value of ${DF}_{i,j,k}$ calculated for supplementary phantom *k* of segment *j* of site *i*;

${\bar{DF}}_{i,j}$ – segment-specific population-average value of *DF*;

${\sigma_{var}}_{i,j}$ – segment-specific individual variability of *DF* in terms of root mean square deviation.

**Fig. 3:**
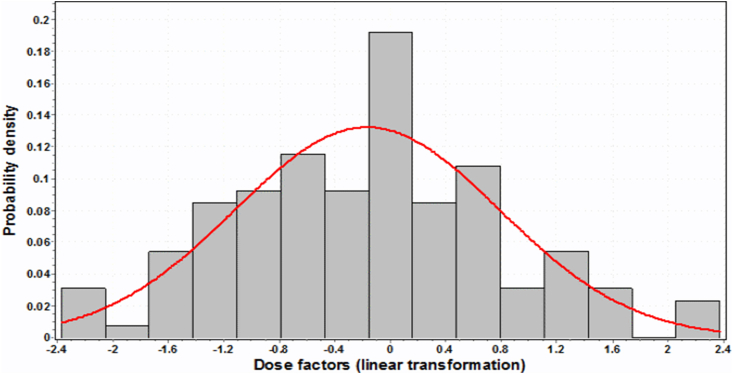
Distribution of linearly transformed dose factors $\tilde{DF}\left(AM\leftarrow CBV\right)$. Solid line is a 3-parameter lognormal fit.

Fig. 3 shows the distribution of the linearly transformed $\tilde{DF}\left(AM\leftarrow CBV\right)$. The distribution looks symmetrical. A similar distribution was observed for $\tilde{DF}\left(AM\leftarrow TBV\right)$. The fact that the shape of the distribution appears symmetrical confirms the suitability of using the uncertainty propagation rule (Eqn. (8)). However, three tests of the best fit (Kolmogorov-Smirnov, Anderson-Darling and Chi-squared) show that a 3-parameter lognormal distribution (shift parameter is necessary to take into account the negative values) demonstrates a better agreement with the data compared to the Gaussian curve. A three-parameter lognormal data fit is shown by the solid curve in Fig. 3.

The lognormal shape of segment-specific *DF* distribution (*LogN*(μ,s)) was propagated to the shape of skeletal-average *DFs* (at the final stage of overall uncertainty calculation); μ and s were calculated according to Eqn (11).

(11) $$\left\{ \begin{array}{c} \mu =\ln\left(DF/\sqrt{\left(\delta^{2}+1\right)}\right)\\ s=\sqrt{\ln\left(\delta^{2}+1\right)} \end{array}\right.$$

#### 4.1.4 Variability of AM distribution among hematopoietic sites

Skeletal-average dose factors are calculated considering the site-specific *AM* fraction (Eqn. (8)). Data on *AM* fraction individual variability [34] were available for adults only [1,2]. Variation of *AM* fraction for adults was on average about 28%, the sites with *AM* fraction >0.1 have a coefficient of variation (CV) < 24%. The variability typical of adults can be roughly used to describe the variability of *AM* fractions for other ages. The data on *AM* fraction for children were taken from Ref. [33]. To account for possible bias, the relative deviation of *AM* fractions reported by Cristy [33] for adults were compared to those given in Ref. [34]. The relative root mean square deviation can be associated with non-excluded systematic error, which should be treated as an additional contributor to overall uncertainty. Table 7 shows the comparison of [33,34] data on the mean *AM* fractions in different hematopoietic sites of adults.

**Table 7:** Comparison of Campbell et al. [34] and Cristy [33] data on the *AM* distribution in different hematopoietic sites of adult human skeleton.

Site	Campbell et al. [34]		Cristy [33]		Assumed *CV* for data of [33]
	Age<65 y		Age 25 y	Age 40 y	
	*AM* fraction	CV	*AM* fraction	*AM* fraction	
Head (Cranium + mandible)	0.062	0.37	0.085	0.084	0.46
Scapulae + ribs + clavicle	0.153	0.17	0.189	0.197	0.27
Sternum	0.018	0.39	0.03	0.031	0.57
Cervical Spine	0.035	0.29	0.037	0.039	0.30
Thoracic Spine	0.175	0.14	0.153	0.161	0.18
Lumbar Spine	0.155	0.16	0.117	0.123	0.33
Sacrum	0.074	0.24	0.094	0.099	0.34
Femora (upper half)	0.059	0.42	0.074	0.067	0.46
Humeri (upper half)	0.036	0.53	0.025	0.023	0.73
Pelvic Bones (Ossa coxae)	0.232	0.13	0.195	0.175	0.29
Average CV of *AM* fractions					0.39

The difference between *AM* fractions in different hematopoietic sites for adults aged 25 and 40 [33] is not large and on average is equal to 6%. Site-specific differences between average *AM* fractions of [33] and *AM* fractions estimated by Campbell et al. [34] range from 8 to 50% (25% on average). The minimum difference (<18%) is typical of cervical and thoracic spine as well as femora (they contain about 27% of skeletal *AM*). The maximum difference (>40%) was observed in data on sternum and humeri (they contain about 5.5% of skeletal *AM*). Overall site-specific uncertainties of *AM* fractions are shown in the last column of Table 7. These site-specific values were applied to phantoms of children. We do not separate the inherent and introduced uncertainties and treat the overall uncertainty as a variability. Skeletal models of children include the sites that are not hematopoietic in adults (radius, tibia and fibula). An average value of *AM* fraction variability (39%) was applied to them.

### 4.2 Introduced uncertainties

#### 4.2.1 Effect of voxel resolution

**Fig. 4:**
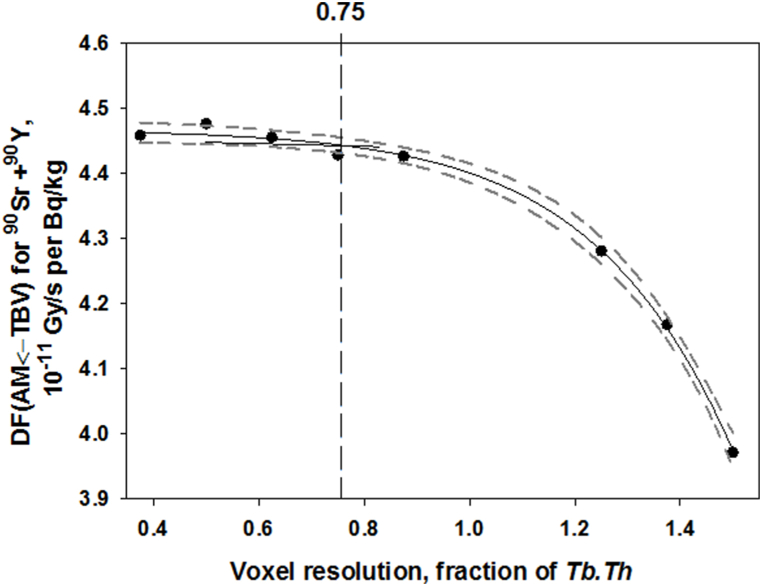
*DF*_*i,j*_*(AM ← TBV)* as a function of voxel resolution. Points are the results of Monte Carlo simulation, solid line is a spline; dashed lines around the spline bound the 90% confidence interval. The vertical line borders the voxel resolution above which the values of *DF*_*i,j*_*(AM ← TBV)* differ from the best estimate (with the minimum resolution).

The use of an insufficient voxel resolution in a computational phantom can distort the geometry and introduce an error in calculation. This is of special importance for the spongiosa microstructure. The range of segment-specific average *Tb.Th* is 75–290 μm [24]. The thickness of individual trabeculae within the spongiosa may vary from 40 to 400 μm [25]. Ideally, the voxel resolution should be less than the minimum size of an object under description. However, <40 μm voxel resolution may result in extremely large input files and could take a significant amount of computer memory and time to execute. Evaluation of *DF*_*i,j*_*(AM ← TBV)* as a function of voxel resolution is a way to define the uncertainty due to voxelization as well as to estimate the optimal voxel resolution (maximum suitable value that does not lead to a bias). Fig. 4 illustrates the dependence of *DF*_*i,j*_*(AM ← TBV)* on voxel resolution. Voxel resolution is represented in terms of *Tb.Th* fraction.

As shown in Fig. 4, the voxel resolutions ≤0.75 × *Tb.Th* do not lead to the bias of *DF*_*i,j*_*(AM ← TBV).* The fitted curve below 0.75 × *Tb.Th* is within the 90% confidence interval of the best *DF* estimate with the minimum voxel resolution (0.38 × *Tb.Th*). The difference between the best *DF* estimate and *DF* obtained with 0.75 × *Tb.Th* is 1%. Therefore, the voxel resolutions ≤0.75 × *Tb.Th* should be used to generate the segment-specific phantoms. If we fulfil this condition, then the relative standard uncertainty introduced by voxelization is equal to 1%.

#### 4.2.2 Stylization of bone geometry

This factor of uncertainty influence was roughly estimated *ad maximum* by combining phantoms (independently of the modeled bone) of different shape and proportion but with similar linear dimensions of a box circumscribed around the models. All models were divided into groups. Criteria of grouping were different for analysis of *DF(AM ← TBV)* and *DF(AM ← CBV).*

*DFs (AM ← TBV)* are shape and size independent and correlate with *BV/TV* only for segments with edges of a box circumscribed around the models >1.1 cm (λ(Q)). In contrast, *DF(AM ← TBV)* of the smallest segments with spongiosa volume, *V*_*s*_, <1 cm^3^ highly correlate with both *V*_*s*_ and spongiosa surface area, SS, (Spearman's correlation coefficients r_s_ > 0.87). Two other groups represent the rest of the models with 1 or 2 dimensions <1.1 cm. Table 8 describes the groups and shows the correlations of *DF(AM ← TBV)* with *BV/TV, V*_*s*_, and *SS*. Only *r*_*s*_ with *p* < 0.05 are shown.

**Table 8:** Description of phantom grouping for analysis of Spearman's correlation (r_s_, *p* < 0.05) of dose factors and potential factors of influence: spongiosa volume – *V*_*s*_; spongiosa surface area –*SS*; bone volume fraction in spongiosa - *BV/TV* (for *DF(AM ← TBV));* cortical thickness *– Ct.Th* (for *DF(AM ← CBV)). δ*_*geom*_ - the uncertainty associated with stylization of bone geometry within the segment groups. *1d*, *2d* and *3d* are 1 dimension, 2 dimensions and 3 dimensions of the circumscribed box, respectively.

Group	Group description	N	*r*_*s*_				δ_geom, %_
*DF(AM ← TBV)*							
			V_s_	SS	BV/TV	SS × BV/TV	
TBV1	*3d* > 1.1 cm	356	0.025^a^	0.051^a^	0.980	–	neglected
TBV2	*V*s *>*1.1 cm^3^, *1d* < 1.1 cm	150	0.389	0.458	0.740	0.893	15
TBV3	*V*_*s*_ >1.1 cm^3^, *2d* < 1.1 cm	116	0.480	0.430	0.654	0.719	13
TBV4	*V*_*s*_*<*1.1 cm^3^	54	0.880	0.874	0.659	0.964	7
*DF(AM ← CBV)*							
			*V*_*s*_	*SS*	*Ct.Th*	*Ct.Th/V*_*s*_	
CBV1	*3d* > 2.8 cm	47	−0.03^a^	0.23^a^	0.953	–	neglected
CBV2	*3d* > 1.5 cm, but some <2.8 cm.	103	−0.747	−0.399	0.631	0.872	15
CBV3	*3d* > 1.1 cm, but some <1.5 cm.	206	−0.626	−0.072	0.701	0.861	10
CBV4	*1d, 2d* or *3d* < 1.1; *V*_*s*_ > 1.6 cm^3^	279	−0.950	−0.689	0.818	0.976	14
CBV5	*1d, 2d* or *3d* < 1.1; 1.6 cm^3^ >*V*_*s*_ > 0.2 cm^3^	39	−0.878	−0.866	0.402	0.78	9
CBV6	*V*_*s*_ < 0.2 cm^3^	26	−0.947	−0.945	0.111	0.949	4

a p > 0.05.

According to Table 8 the correlation of *DF(AM ← TBV)* and both *V*_*s*_ and *SS* is absent in the TBV1 group (all dimensions>1.1 cm) and the influence of shape and size can be ignored. In other groups, the correlations with all factors of influence are notable. This means that in order to assess the influence of shape and size, we should remove the influence of *BV/TV*.

**Fig. 5:**
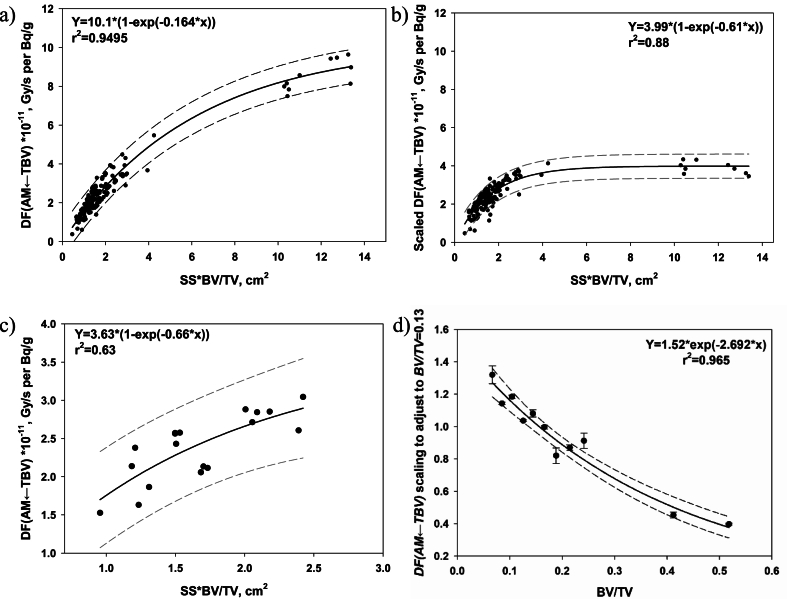
Scaling of the ^90^Sr *DF(AM←TBV)* of group TBV2: a) and b) illustrate the dependence of raw and scaled data, respectively, on SS × BV/TV; c) reference function for data obtained using the models with *BV/TV* = 0.13; d) scaling factor function for recalculation of data with different *BV/TV* to fixed one (*BV/TV* = 0.13). Solid lines are the data smoothing; dashed lines bound the 90% prediction intervals; dotted lines are 90% confidence boundaries.

Note, that the *DF* correlation with combined parameter, *SS×BV/TV*, is stronger than the correlations with each of the multipliers separately. The dependence of *DF(AM ← TBV)* on *SS×BV/TV* in each group can be fitted by exponential rise to maximum (Eqn. (12)) (an example for TBV2 is shown in Fig. 5a).

(12) $${DF}^{small}\left(AM\leftarrow \;TBV\right)=a\times \left(1-e^{-b\times \left(SS\times \frac{BV}{TV}\right)}\right)\text{,}$$

where ${DF}^{small}\left(AM\leftarrow \;TBV\right)$ are *DFs* typical of the models with all dimensions <1.1 cm; *a* and *b* are scale and shape parameters of the function, with values that vary for each of the different sample groups.

For each of the groups, we recalculated the data to a given (reference) *BV/TV* value (Fig. 5b). For this purpose the *DF(AM ← TBV)* obtained with the largest set of models with equal *BV/TV* were selected as a reference data set. In TBV2 and TBV4 the reference *BV/TV* was equal to 0.13; in TBV3, it was 0.17. The reference data were fitted by the function (Eqn. (12)) as shown in Fig. 5c. All primary data on *DF(AM ← TBV)* within the groups were normalized to the corresponding values predicted by the reference function. The obtained ratios were well-fitted by exponential decrease with BV/TV increase (see example in Fig. 5d). Obtained ratios as a function of *BV/TV* were used as a scaling factor to recalculate all group-specific *DF(AM ← TBV)* to the reference *BV/TV* (Fig. 5b). Fig. 5 (a,b,c,d) illustrates the stage of the scaling on the example of group TBV2.

Comparing Fig. 5a and b, note that the scaling reduces the range of *DF(AM←TBV)* values by a factor of 2.6. Nevertheless, the difference in data scattering is not so evident. The analysis of residuals gives the values of 18% for the dispersion of the raw data relative to the fitting curve and 15% for the dispersion of the scaled data relative to the fitting curve. This value corresponds to the impact of shape and size variation uncertainty on the *DF* uncertainty in TBV2. Similar analysis for TBV3 and TBV4 demonstrates a more explicit effect of scaling: the relative dispersion decreases by factors of 1.6 and 2.3, respectively. The values of uncertainties associated with shape and size for different groups are shown in Table 8. A maximum δ_geom_ = 15% was found for TBV2.

A similar approach was used for the estimation of δ_geom_ for *DF(AM←CBV).* However, here the data were clustered into 6 groups. Only data on models with edges of a box circumscribed around the spongiosa models >2.8 cm were insensitive to size and shape but highly correlated (*r*_*s*_ = 0.953) with *Ct.Th* (Table 8). The combined parameter, for which *DF(AM←CBV)* correlation is stronger than the correlations with the separate factors of influence, is *Ct.Th/V*_*s*_. Estimates of group-specific δ_geom_ of *DF(AM←CBV)* are shown in Table 8 and vary from 4% to 15%.

#### 4.2.3 The assumption of cortical thickness uniformity

Our calculations, as well as the calculations for phantoms of the University of Florida series [18,19], assumed a homogeneous cortical layer of uniform thickness. According to Table 8, cortical thickness is the leading factor of influence for models with spongiosa volume >1.6 cm^3^. The effect of cortical layer non-uniformity on *DF(AM* ← *CBV)* was tested with 4 sets of cubic spongiosa models. Two sets have a spongiosa volume exceeding 1.6 cm^3^ (with edge length L equal to 1.2 and 2.1 cm). Each of the sets consists of 13 models. The first set (basic) has an equally-thickened cortical layer at each face. Twelve additional models each have the same average *Ct.Th* but with *Ct.Th* at different faces varied randomly. Table 9 presents the example of *Ct.Th* randomization and corresponding *DF(AM* ← *CBV)* for a set of models generated based on the cubic spongiosa model with *L* = 2.1 cm and mean *Ct.Th=*0.035 cm.

**Table 9:** An example of the results of random sampling of cortical thickness at different faces of the cubic spongiosa model (*L* = 2.1 cm and mean *Ct.Th=*0.035 cm). *F*_*n*_ is a cube face, where *n* is a number of the face. RMSD – root mean square deviation from the basic model.

Model number	Ct.Th × 10^−2^, cm						DF (AM←CBV), × 10^−11^ (Gy s^−1^)/(Bq g^−1^)
	*F*_*1*_	*F*_*2*_	*F*_*3*_	*F*_*4*_	*F*_*5*_	*F*_*6*_	
1 (*basic*)	3.5						0.996
2	3.8	3.2	4.3	2.9	2.1	4.7	1.010
3	3.7	2.6	4.6	3.4	2.6	4.1	0.948
4	3.7	3.1	3.8	4.4	1.3	4.7	0.956
5	2.4	4.1	5.0	4.9	4.2	0.4	0.971
6	3.4	2.9	4.7	1.5	3.7	4.8	0.962
7	0.2	4.9	4.7	1.9	4.7	4.6	1.000
8	3.3	3.6	3.4	1.1	4.8	4.8	0.963
9	3.8	4.1	1.0	2.9	4.6	4.6	0.981
10	2.2	4.3	4.2	4.8	2.8	2.7	0.973
11	3.6	3.7	1.5	4.0	3.3	4.8	0.998
12	3.4	3.6	4.6	3.2	1.5	4.7	0.981
13	3.0	3.2	3.6	3.5	3.9	3.6	0.993
Relative RMSD	33%						3%

As can be seen from Table 9, the 33% side-to-side variation of *Ct.Th* results in an insignificant variability of *DF* (AM←CBV); at least for mean *Ct.Th=*0.035 cm. Table 10 provides a summary of the relative RMSD for different *Ct.Th* and spongiosa dimensions.

**Table 10:** Relative RMSD for different spongiosa dimensions (*L* – the edge length of cubic spongiosa model) and *Ct.Th*.

*L,* cm	Relative RMSD of *DF*(AM←CBV), %	
	*Ct.Th* = 0.035 cm	*Ct.Th* = 0.084 cm
2.1	3	7
1.2	5	7
1	2	7
0.7	2	6

As shown in Table 10, the relative RMSD of *DF* (AM←CBV) for the most typical values of *Ct.Th* is 7% or lower. Therefore, 7% can be conservatively assumed as an uncertainty introduced by the assumption of cortical thickness uniformity.

#### 4.2.4 Restriction of bone geometry

Several restricted models have been used to create segment-specific phantoms (e.g., skull bones, ribs). Restrictions on bone geometry can lead to an underestimation of energy deposition in *AM* due to electron emission from the neighboring parts of the bone adjacent to the segment.

Volchkova et al. [40] reported in detail the study of the effect of bone marrow exposure to cross-fire from the adjacent bones for segments of different size, shape and *BV/TV*. It has been shown that if the area of the spongiosa surface (*SS*) is > 6 cm^2^, then the crossfire effect is negligible. For a smaller *SS,* the extension of the linear dimensions of the spongiosa bone by 2 mean electron path lengths (*SS*_*ext*_) results in a *DF*_*i,j*_ (AM←TBV) increase by a factor of ${\left({SS}_{ext}\;/\text{SS}\;\right)}^{0.28}$. These values vary within the range 1.03–1.21 and are used as adjustment coefficients for the *DF*_*i,j*_ (AM←TBV). The relative standard uncertainty of the adjustment coefficient is 5%.

## 5 Discussion

### 5.1 Analytical description of dose factors

The bone marrow dosimetry is complicated and laborious due to the large number of bones in the skeleton, each of which needs to be described considering a complex three-dimensional micro and macro architecture. The problem can be simplified by an analytical description of *DF* dependences on the parameters of computational phantoms. For example, Eqn. (13) describes *DF(AM ← TBV)* as a function of *SS×BV/TV*. However, scale and shape parameters of Eqn. (13) would be different for different data groups. This makes the generic equation inconvenient to use. Moreover, the uncertainty of the *DF* predictions resulting from the use of this equation is too large (>15%). Therefore, it is preferable to obtain *DF*_*i,j*_*(AM ← TBV)* with Monte Carlo simulations, at least for models of small-sized bone segments.

**Fig. 6:**
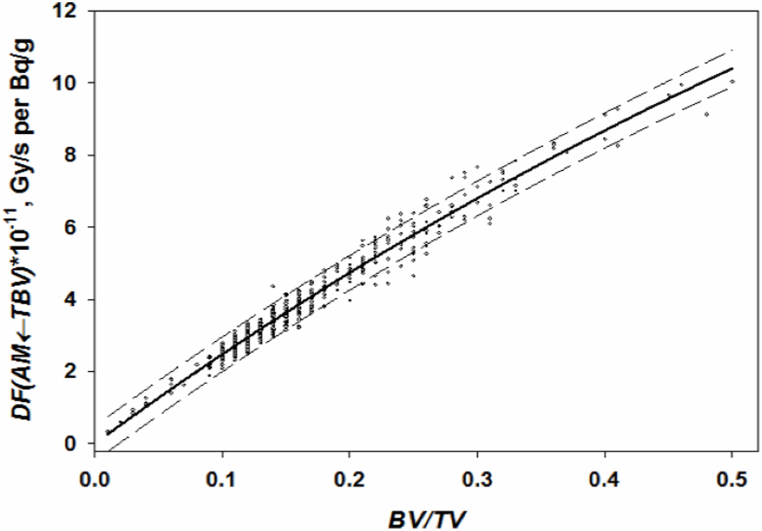
Dependence of *DF*_*j*_*(AM ← TBV)* calculated for ^90^Sr on *BV/TV* for bone segment phantoms with spongiosa linear dimensions >0.46 cm. Solid line is a fitted function of exponential rise to maximum. Points are the results of calculations. Dashed lines bound the 90% prediction interval.

According to Shishkina et al. [26], an analytical approach can be applied to phantoms with all spongiosa linear dimensions > $2\times \lambda \left(\bar{E}\right)$ (∼0.46 cm). For such segments, the influence of size and shape is not decisive. *DF(AM ← TBV)* is defined only by spongiosa density, which is proportional to the *BV/TV* parameter. Fig. 6 demonstrates the dependence of *DF*_*i,j*_*(AM ← TBV)* for ^90^Sr on *BV/TV*. The dependence is well fitted by a function of exponential rise to maximum (Eqn. (13); *r*^2^ = 0.964). The function is defined on the interval of *BV/TV* = [0.01, 0.52].

(13) DFi,j(AM←TBV)={Sr90:28(1−e−0.897BVTV)Sr89:13.9(1−e−1.06BVTV),(10−11GysperBqgofSr90)

The residual analysis demonstrates a decrease of relative residuals from 15% to 6.5% with a *BV/TV* increase from 0.01 to 0.52. As ${DF}_{i,j}\left(AM\leftarrow TBV\right)$ decreases, the relative RMSD increases. This relationship can be formulated as follows: $\delta_{{DF}_{i,j}\left(AM\leftarrow TBV\right)}=0.063+0.1e^{-0.373{DF}_{i,j}\left(AM\leftarrow TBV\right){∙10}^{11}}$ [26] and varies from 5% to 15%. On average, $\delta_{{DF}_{i,j}\left(AM\leftarrow TBV\right)}$ takes on the value 7.4% (when *BV/TV* = 0.265).

**Fig. 7:**
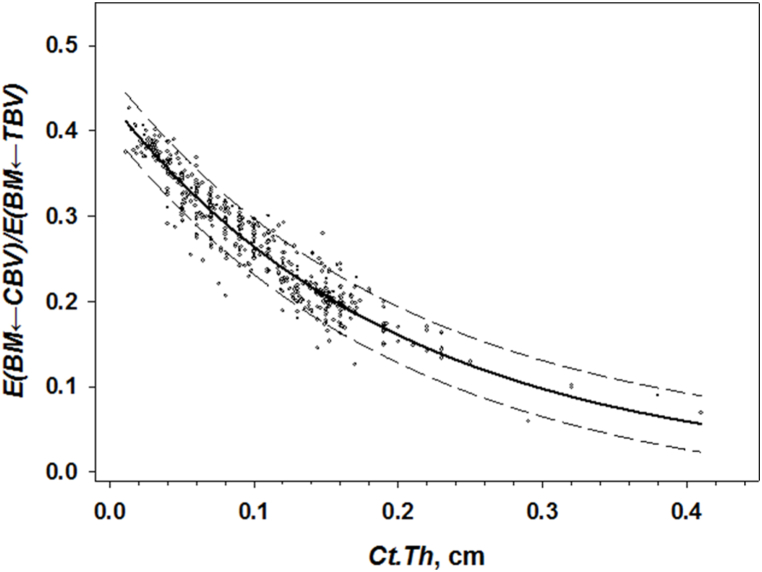
Segment-specific ^90^Sr E(BM←CBV) to E(BM←TBV) ratio as a function of *Ct.Th* for phantoms with linear dimensions >0.46 cm. Solid line is a fitted exponential function. Points are the results of calculations. Dashed lines bound the 90% prediction interval.

If electrons are emitted in *CBV,* then energy deposition in *BM* decreases exponentially with an increase of *Ct.Th (*$E_{i,j}\left(BM\leftarrow TBV\right)$ *=a∙e*^*-b∙Ct.Th*^) due to self-attenuation (assuming all other model parameters being equal). However, additional factors (such as shape and size of spongiosa) may influence energy deposition in bone marrow, at least for models with all linear dimensions <2.8 cm (Table 8). Nevertheless, it is possible to reduce the impact of these additional factors by considering that calculations of absorbed energy in *BM* from both *TBV* and *CBV* were performed in parallel using the same phantom (the same $\frac{BV}{TV},SS\;\text{and}\;Vs$). Then the following approximation can be used (Eqn. (14)).

(14) $$\frac{E\left(КM\leftarrow CBV\right)}{E\left(КM\leftarrow TBV\right)}=\frac{f_{CBV}\left(\frac{BV}{TV},SS,Vs,Ct.Th\right)}{f_{TBV}\left(\frac{BV}{TV},SS,Vs\right)}\approx F_{c}\left(Ct.Th\right)$$

where $f_{CBV}\left(\frac{BV}{TV},SS,Vs,Ct.Th\right)$ is a function describing the dependence of E(AM←CBV) on a set of phantom characteristics; $f_{TBV}\left(\frac{BV}{TV},SS,Vs\right)$ is a function describing the dependence of E(AM←TBV) on a set of phantom characteristics; $F_{c}\left(Ct.Th\right)$ is an approximation of the energy deposition ratios as a function of *Ct.Th* only. Fig. 7 shows an example of fitting the calculated energy deposition ratios to the exponential function of *Ct.Th*. The dependence is well fitted (*r*^2^ = 0.93) by exponential function (Eqn. (15)) as follows:

(15) $$\frac{E\left(BM\leftarrow CBV\right)}{E\left(BM\leftarrow TBV\right)}=0.435\times e^{-4.98\times Ct.Th}$$

The function is defined on the interval of *Ct.Th* = [0.01, 0.41] cm. The residual analysis demonstrates that relative residuals vary from 4% to 20%. On average (*Ct.Th* = 0.21 cm), $\delta_{E\left(КМ\leftarrow CBV\right)/E\left(КМ\leftarrow TBV\right)}$ = 10%.

The ratio (Eqn. (15)) allows us to express the ${DF}_{i,j}\left(AM\leftarrow CBV\right)$ in terms of ${DF}_{i,j}\left(AM\leftarrow TBV\right)$ with adjustment for *Ct.Th* (Eqn. (16)).

(16) DFi,j(AM←CBV)={Sr90:DFi,j(AM←TBV)×0.435e−4.98Ct.Th×VCBVVTBVSr89:DFi,j(AM←TBV)×0.431e−6.915Ct.Th×VCBVVTBV,GysperBqgofSr90)

Using Eqn. (13), Eqn (16) can be reformulated as Eqn. (17)

(17) DFi,j(AM←CBV)={Sr90:12.2e−4.98Ct.Th(1−e−0.897BVTV)VCBVVTBVSr89:6e−6.915Ct.Th(1−e−1.06BVTV)VCBVVTBV,×10−11GysperBqgofSr90

The overall uncertainty of ${DF}_{i,j}\left(AM\leftarrow CBV\right)$ (calculated with Eqn. (17)) depends on both *BV/TV* and *Ct.Th*. The uncertainty is calculated as follows (Eqn. (18)):

(18) $$\delta_{{DF}_{i,j}\left(AM\leftarrow CBV\right)}=\sqrt{\delta_{E\left(AМ\leftarrow CBV\right)/E\left(AМ\leftarrow TBV\right)}^{2}+\delta_{{DF}_{i,j}\left(AM\leftarrow TBV\right)}^{2}}$$

The $\delta_{{DF}_{i,j}\left(AM\leftarrow CBV\right)}$ is below 13% for most combinations of *BV/TV* and *Ct.Th* typical of human bones. Thus, the uncertainty introduced in the analytical description of both ${DF}_{i,j}\left(AM\leftarrow TBV\right)$ and ${DF}_{i,j}\left(AM\leftarrow CBV\right)$ for bone segment phantoms with spongiosa linear dimensions > $2\times \lambda \left(\bar{E}\right)$ is comparable with the uncertainty introduced by the geometric approximations used for the Monte Carlo simulation of electron-photon transport. Thus, the analytical approach can be useful for simplifying routine calculations.

### 5.2 Summary of errors and uncertainties

Table 11 summarizes the contributions of different factors of influence on dose factors calculated with bone segment phantoms. The main contributor to ${DF}_{i,j}\left(AM\leftarrow TBV\right)$ uncertainty of bone segment phantoms with linear dimensions <1.1 cm is the shape stylization. For ${DF}_{i,j}\left(AM\leftarrow CBV\right)$, maximum uncertainties are introduced by stylization of bone segment phantoms with spongiosa linear dimensions within the range of 1.1–2.8 cm. According to Table 11, the model approaches do not introduce errors of more than 15% for ${DF}_{i,j}\left(AM\leftarrow TBV\right)$ and 17% for ${DF}_{i,j}\left(AM\leftarrow CBV\right)$. This is less than the errors due to inherent variability of perhaps >60%.

**Table 11:** Summary of the contributions of different factors of influence on dose factors calculated for bone segment phantoms; *1d*, *2d* and *3d* are 1 dimension, 2 dimensions and 3 dimensions of the circumscribed box, respectively.

Source of uncertainty	Standard relative uncertainty (mean or the range of CV), %	
	${DF}_{i,j}\left(AM\leftarrow TBV\right)$	${DF}_{i,j}\left(AM\leftarrow CBV\right)$
*Introduced by modeling approaches*		
shape stylization depend on bone dimensions		
*3d* > 2.8 cm	–	–
2.8 cm > *3d* > 1.1 cm	–	10–15
*3d* < 1.1 cm	7–13	4–14
voxelization	1	0
model restriction	0–7	0
uniformity of cortical thickness	0	7
*Individual variability of bone characteristics*		
micro and microarchitecture	20 [5–60]^a^	
chemical composition	4	
bone density depend on bone dimensions		
*3d*> $2\times \lambda \left(\bar{E}\right)$	4	–
other	6	13

a mean variability and the range of possible values depend on variability of bone-specific micro and macro-architecture.

For models with linear dimensions >0.46 cm, the uncertainties introduced by the analytical approach (Eqns. (13), (17))) approximate those uncertainties introduced by approaches used in direct Monte Carlo simulation. Therefore, the SPSD method suggests simplifying the modeling of “large” bone segments with the use of Eqns. (13), (17).

The individual bone segments are not of practical interest, but the skeletal-average dose factor (Eqn. (7)) and its uncertainty (Eqn. (8)) are. This uncertainty depends on the variability of active marrow distribution between the skeletal sites (Table 7), which is on average ∼ 40%. This variability of active marrow distribution is the main contributor to overall variability, while the stylization, on average, introduces about 20%.

### 5.3 Shared, unshared and overall uncertainties of skeletal-average DFs

Contemporary epidemiological studies take into account both radiation doses and corresponding uncertainties that may affect the risk estimate uncertainty [23]. Uncertainties in individual dose estimates should be described as shared and unshared uncertainties. Shared uncertainties arise because, for example, of imprecision of a population-average estimate. This uncertainty would be the same for a large group of cohort members. Unshared uncertainties can arise, for example, because of the specific anatomy of an individual.

The dose factors will be incorporated into the stochastic version of TRDS (TRDS-2016MC). TRDS-2016MC uses a two-dimensional Monte Carlo method [23] accounting for both shared uncertainties and independent unshared uncertainties. Therefore, the uncertainties obtained in the framework of the study were classified in this manner. The introduced uncertainty for skeletal-average dose factor can be identified as being shared; the uncertainty due to variability can be identified as being unshared. The shared uncertainty component of the skeletal-average DF(AM←s) (for both TBV and CBV) is 3–4 times less than the unshared component. Table 12 demonstrates an example of uncertainty components calculated for dose factors of an adult male (Supplemental material S1).

**Table 12:** Uncertainties of^90,89^Sr dose factors of an adult male in terms of CV; 90% CI calculated assuming a lognormal distribution.

DF	Mean [90% CI], $\times 10^{-11}\frac{Gy}{s}per\frac{Bq}{g}$	Shared uncertainty, %	Unshared uncertainty, %	Overall uncertainty, %
DF(AM←TBV)				
^90^Sr	4.20 [2.4–6.2]	6	25	26
^89^Sr	2.33 [1.5–3.4]	7	25	26
DF(AM←CBV)				
^90^Sr	1.54 [0.8–2.6]	11	36	38
^89^Sr	0.74 [0.4–1.3]	9	37	38

Overall uncertainties of *DF(AM ← TBV)* and *DF(AM ← CBV)* for different ages are below 55% and 63%, respectively. Note that the younger the age, the greater the shared uncertainty of DF(AM←TBV) is, because the number of small segments increases and the impact of stylization uncertainty increases too. The maximum shared uncertainty typical of DF(AM←TBV) of newborn and 1-year-old was about 14%. The maximum shared uncertainty of DF(AM←CBV) was calculated for 15-year-old (CV = 16%). At this age the dimensions of most bone segments are in the range of 1.1–2.8 cm. According to Table 11, the greatest contributor to uncertainty for such segments is due to stylization. In any case, the shared uncertainty of DF(AM←CBV) does not exceed 16%. The unshared uncertainty component is comparable with the mean uncertainty of bone marrow distribution of AM among the hematopoietic skeletal sites and to a lesser extent supplemented by the uncertainty of bone dimension variability. The unshared component of uncertainty is inherent and can be applied for different methods of beta-particle dosimetry of the bone marrow for incorporated strontium isotopes, such as models based on chord-length distributions [14] or image-based skeletal dosimetry models [18,19]. For example, estimates with the image-based phantoms are also not free of uncertainty associated with the variability of bone density, chemical composition and bone micro- and macro architecture. Moreover, the image-based computational phantoms also assumed cortical layer uniformity, and our estimates of 7% of shared error could be applied to the results of image-based method computations. The image-based methods used for the creation of computational phantoms are free from the error associated with stylization. However, this advantage may be offset by the fact that the phantoms are based on images from a limited number of individuals. We suppose that the representativeness error may be comparable to the stylization error of the SPSD approach.

Within the framework of the current study the skeletal-average DFs were calculated assuming a homogeneous radionuclide distribution in TBV and CBV of the entire skeleton to harmonize the dosimetric and biokinetic models [42,43]. However, the SPSD dosimetric approach readily accounts for the ^90^Sr redistribution between skeletal regions using site-specific DFs (see Supplemental materials S1).

### 5.4 The use of the obtained results

The SPSD model limits the source tissues to only the trabecular and cortical volumes. Due to their half-lives, short-lived beta emitters (e.g., ^153^Sm - 1.93 d; ^177^Lu - 6 d) do not have time to integrate into the bone volume. Accurate dose calculations for these short-lived radionuclides would require the inclusion of bone surfaces as separate bone compartments in the model. In other words, the presented model was elaborated for Sr isotopes and would require modification to evaluate surface-deposited beta emitters as well as alpha emitters.

However, the uncertainties of dosimetric modeling associated with the variability of physical-chemical parameters (e.g., density, chemical composition) obtained in this work can be extended to other beta emitters. The variability of the AM distribution among the hematopoietic sites would have the same effect on the uncertainty of dose estimates for different radionuclides.

The current study is important for analyzing the radiation effects of chronic exposure of AM. The investigation of radiation risks of leukemia is carried out in cohorts of the exposed population in the Southern Urals. Proper characterization of the uncertainty is important in assessing the statistical significance of the risk estimates and understanding the range of risks, which can be important in risk management. Dose uncertainties have to be considered in the assessment of radiation risk reliability.

On average, about 80% of the accumulated absorbed dose in *AM* of the studied population results from internal exposure [44,45] mainly due to incorporated strontium isotopes. Therefore, special attention is paid to the uncertainty of this dose component. The current study is an important component of a complex effort to estimate uncertainty for bone marrow dosimetry. Other important contributors to dose uncertainty are individual intake function and radionuclide retention function (Eqn. (1)), which were described by Tolstykh et al. [[8], [9], [10],^12^].

The results obtained will be incorporated into the stochastic version of the Techa River Dosimetry System (TRDS-2016MC). The unshared uncertainty will be simulated assuming a lognormal distribution (Eq. (11)). The shared uncertainty component can be approximated by a normal distribution.

## 6 Conclusions

The study was motivated by the ongoing epidemiological investigations of the effects of chronic exposure of a population residing in the radioactively contaminated territories of the Southern Urals. On average, the contribution of internal exposure to the total active marrow dose of the exposed population was about 80%, where the largest component was associated with incorporated strontium isotopes. Therefore, the accurate estimation of all uncertainty contributors, including the uncertainty of dosimetric modeling is a key issue.

Summarizing the results presented, it should be concluded that a comprehensive stochastic parametric skeletal dosimetry (SPSD) approach is useful for assessing the active marrow dose from bone-seeking beta-emitting radionuclides along with their corresponding uncertainties. The dose factors *DF(AM* ← *TBV)* and *DF(AM* ← *CBV)* provide direct conversion of ^90^Sr and ^89^Sr concentration in the source regions (*TBV* and *CBV*) to the dose rate in the target region (*AM*).

The inherent variability (unshared uncertainty) of skeletal-average dose factors is about 40–50%. The model explicitly accounts for the *AM* distribution in the skeleton, the variability of which is the main contributor to unshared uncertainty. One more important contributor to inherent uncertainty of dose factors is the individual variability of bone micro and macro dimensions. The influence of the variability on *DFs* depends on bone size, with an average CV of 20%. However, in relatively small bone segments the CV may reach 60%. The uncertainty of *DF*s due to individual variability of bone chemical composition is less than 4%. Uncertainties of *DF(AM ← s)* due to the individual variability of bone density are segment specific and depend on the spongiosa size. The uncertainties of *DF(AM ← TBV)* and *DF(AM ← CBV)* of bone segment phantoms with linear dimensions of spongiosa less than 2 mean electron pathlengths (continuous slowing-down approximation) are equal to 6% and 13%, respectively.

The uncertainty components introduced by the SPSD approach (shared uncertainty) do not exceed 16% and mainly depend on the error of bone shape stylization. About 7% of *DF(AM ← CBV)* uncertainty is introduced by the assumption of uniformity of cortical thickness. Thus, the uncertainty introduced by modeling is much less than from individual variability. Overall uncertainties of *DF(AM ← TBV)* and *DF(AM ← CBV)* are below 55% and 63%, respectively.

The inherent components of overall uncertainty, estimated in the current study, can be extended to the dosimetric modeling based on different methods of bone marrow dosimetry for incorporated strontium isotopes.

The dose factor for bone segment phantoms with spongiosa linear dimensions > $2\times \lambda \left(\bar{E}\right)$ can be calculated analytically. The uncertainties introduced in the analytical description of both ${DF}_{i,j}\left(AM\leftarrow TBV\right)$ and ${DF}_{i,j}\left(AM\leftarrow CBV\right)$ are comparable to those introduced by stylization of bone geometry. Thus, the analytical approach can be useful for simplifying routine calculations for a large proportion of bone segments.

The results obtained will be incorporated into the stochastic version of the Techa River Dosimetry System (TRDS-2016MC) that provides multiple realizations of the annual doses for each cohort member to obtain both a central estimate of the individual dose and information on the dose uncertainty.

## Funding statement

Elena Shishkina, Pavel Sharagin, Evgenia Tolstykh and Marina Degteva were supported by Federal Medical-Biological Agency of Russia Contract N◦ 27.501.19.2 in the framework of Russian Federal Targeted Program “Provision of nuclear and radiation safety for the period 2016–2020 and for the period up to 2035”. https://xn----btb4bfrm9d.xn--p1ai/and US 10.13039/100000015Department of Energy, Project Joint Coordinating Committee for Radiation Effect Research (JCCRER) dose reconstruction for the Urals. https://www.energy.gov/ehss/international-health-studies-and-activities.

Michael Smith and Bruce Napier were supported by 10.13039/100011661PNNL Contract DE-AC05-76RL01830, (US 10.13039/100000015Department of Energy), Project JCCRER DOSE RECONSTRUCTION FOR 10.13039/501100000725THE URALS, Budget and Reporting Number HS0240030, https://www.energy.gov/ehss/international-health-studies-and-activities.

The funders had no role in study design, data collection and analysis, decision to publish, or preparation of the manuscript.

## Data availability statement

Data will be made available on request.

## CRediT authorship contribution statement

**Elena A. Shishkina:** Writing – original draft, Formal analysis, Conceptualization. **Pavel A. Sharagin:** Writing – review & editing, Software, Investigation, Formal analysis. **Evgenia I. Tolstykh:** Investigation, Formal analysis. **Michael A. Smith:** Writing – review & editing, Software, Investigation, Formal analysis. **Bruce A. Napier:** Investigation, Formal analysis. **Marina O. Degteva:** Formal analysis, Conceptualization.

## Declaration of competing interest

The authors declare that they have no known competing financial interests or personal relationships that could have appeared to influence the work reported in this paper.
