# Supplementary material for: Uncertainty of stochastic parametric approach to bone marrow dosimetry of 89,90Sr
Source: Heliyon. 2024 Feb 10;10(4):e26275. doi: 10.1016/j.heliyon.2024.e26275 (PMC10900932; doi:10.1016/j.heliyon.2024.e26275)
Supplement: Multimedia component 1 [file mmc1.docx]

**Supplementary materials S1**

**The tables describe the computational phantom of adult male and dose factor calculated for hematopoietic bone segments, sites and the skeleton**

The document contains the parametric description of bone segment phantoms (Tab. S1.1) and the results of dose factor calculations (Tab. S1.2).

The bone segment shapes in Tab. S1.1 indicated graphically as follows:

- – rectangular parallelepiped; linear dimensions are H×a×b (height, length and width);
- –cylinder; linear dimensions are H×a×b (height, major axis and minor axis)
- – deformed cylinder; linear dimensions are H×a×b×c×d (height, major and minor axis of one base and major and minor axis of another base)
- – triangulare prism; linear dimensions are H×a×b (height, base and legs of isosceles triangle)
-
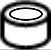
 – tube-like phantom, describes as two cylinders nested one in the other, spongiosa is located between the cylinders; linear dimensions are H×a×b (height, outer and inner diameter or the round base)

Variability of the linear dimensions for full-sized bone segment dimensions were estimated from literature-derived data on individual variability. Some of dimensions were restricted (for example, the ribs length was taken as 30 mm, which is 3 times larger than maximum ^90^Sr+^90^Y electron pathlength). Restricted dimensions of bone segment phantoms not vary in the SPSD model. *Ct.Th* is a cortical thickness; *Tb.Th* is the trabecular thickness; *Tb.Sp* is the trabecular separation; *BV/TV* is the bone volume fraction of spongioza.

**Table S1.1. Parameters of bone phantom segments of different shape, *Sh*, for adult male represented in terms of population average, M (mm), and individual variability, CV (%), or the range of possible values (min-max).**

| Site | Segment | *Sh* | h | | a | | b | | c | | d | | *Ct.Th* | | *BV/TV* | | *Tb.Th* | | *Tb.Sp* | |
| --- | --- | --- | --- | --- | --- | --- | --- | --- | --- | --- | --- | --- | --- | --- | --- | --- | --- | --- | --- | --- |
|  |  |  | M | CV | M | CV | M | CV | M | CV | M | CV | M | CV | M | min-max | M | CV | M | CV |
| Femur | Neck |  | 30 | 13 | 36 | 6 | 32 | 4 |  |  |  |  | 1.9 | 5 | 0.17 | 0.14-0.22 | 0.19 | 19 | 0.78 | 13 |
| Femur | Trochanter area |  | 43 | 26 | 66 | 6 | 44 | 6 | 30 | 7 | 30 | 7 | 2.3 | 15 | 0.11 | 0.08-0.13 | 0.136 | 65 | 0.99 | 20 |
| Humeri | Proximal end |  | 28 | 12 | 56 | 5 | 56 | 5 | 25 | 15 | 25 | 15 | 1.1 | 18 | 0.06 | 0.01-0.13 | 0.1 | 18 | 2.37 | 25 |
| Ribs | 1, 2 |  | 17 | 12 | 30 |  | 7 | 14 |  |  |  |  | 0.7 | 38 | 0.12 | 0.05-0.25 | 0.15 | 12 | 0.82 | 11 |
| Ribs | 5-8 |  | 14 | 14 | 30 |  | 8 | 13 |  |  |  |  | 0.7 | 38 | 0.12 | 0.05-0.25 | 0.15 | 12 | 0.82 | 11 |
| Ribs | 3, 4, 9, 10 |  | 13 | 8 | 30 |  | 7 | 14 |  |  |  |  | 0.7 | 38 | 0.12 | 0.05-0.25 | 0.147 | 12 | 0.82 | 11 |
| Ribs | 11, 12 |  | 11 | 18 | 30 |  | 6 | 17 |  |  |  |  | 0.7 | 38 | 0.12 | 0.05-0.25 | 0.15 | 12 | 0.82 | 11 |
| Sacrum | Body 4-5 |  | 36 | 9 | 28 | 11 | 8.5 | 13 |  |  |  |  | 1.5 | 8 | 0.15 | 0.1-0.19 | 0.1 | 13 | 0.6 | 16 |
| Sacrum | Body 1 |  | 30 | 7 | 40 | 11 | 24.5 | 10 |  |  |  |  | 1.5 | 8 | 0.15 | 0.1-0.19 | 0.1 | 13 | 0.6 | 16 |
| Sacrum | Body 2-3 |  | 46 | 8 | 28.7 | 11 | 15 | 9 |  |  |  |  | 1.5 | 8 | 0.15 | 0.1-0.19 | 0.1 | 13 | 0.6 | 16 |
| Sacrum | Pedicle 1 |  | 13.9 | 14 | 23.7 | 15 | 15.3 | 11 |  |  |  |  | 1.5 | 8 | 0.15 | 0.1-0.19 | 0.1 | 13 | 0.6 | 16 |
| Sacrum | Pedicle 2 |  | 14.2 | 14 | 25 | 11 | 13.6 | 17 |  |  |  |  | 1.5 | 8 | 0.15 | 0.1-0.19 | 0.1 | 13 | 0.6 | 16 |
| Sacrum | Pedicle 3 |  | 13.9 | 14 | 18.3 | 11 | 13.2 | 14 |  |  |  |  | 1.5 | 8 | 0.15 | 0.1-0.19 | 0.1 | 13 | 0.6 | 16 |
| Sacrum | Pedicle 4 |  | 13.9 | 14 | 14.5 | 11 | 11.2 | 18 |  |  |  |  | 1.5 | 8 | 0.15 | 0.1-0.19 | 0.1 | 13 | 0.6 | 16 |
| Sacrum | Sacral ala 1 |  | 30 | 13 | 20 | 10 | 42 | 13 |  |  |  |  | 1.5 | 8 | 0.15 | 0.1-0.19 | 0.1 | 13 | 0.6 | 16 |
| Sacrum | Sacral ala 2 |  | 26 | 15 | 23 | 17 | 25 | 8 |  |  |  |  | 1.5 | 8 | 0.15 | 0.1-0.19 | 0.1 | 13 | 0.6 | 16 |
| Sacrum | Sacral ala 3-4 |  | 19 | 16 | 38.5 | 15 | 18 | 9 |  |  |  |  | 1.5 | 8 | 0.15 | 0.1-0.19 | 0.1 | 13 | 0.6 | 16 |
| Pelvis | Iliac crest |  | 11 | 15 | 30 |  | 13 | 9 |  |  |  |  | 1 | 30 | 0.19 | 0.11-0.25 | 0.13 | 15 | 0.6 | 20 |
| Pelvis | Iliac ala |  | 9.5 | 31 | 30 |  | 30 |  |  |  |  |  | 1 | 30 | 0.19 | 0.11-0.25 | 0.13 | 15 | 0.6 | 20 |
| Pelvis | Iliac dorsal segment |  | 19 | 16 | 30 |  | 30 |  |  |  |  |  | 1 | 30 | 0.19 | 0.11-0.25 | 0.13 | 15 | 0.6 | 20 |
| Pelvis | Pubis ramus superior (lower) |  | 32 | 19 | 15 | 20 | 29 | 20 |  |  |  |  | 0.7  1.5 | 30  12 | 0.17 | 0.12-0.23 | 0.29 | 10 | 1.0 | 12 |
| Pelvis | Pubis ramus superior (upper) |  | 51.2 | 8 | 14.5 | 20 | 16 | 20 |  |  |  |  | 0.7  1.5 | 30  12 | 0.17 | 0.12-0.23 | 0.29 | 10 | 1.0 | 12 |
| Pelvis | Pubis ramus inferior |  | 47 | 17 | 16 | 25 | 22 | 23 | 26 | 23 | 14 | 36 | 0.5 | 30 | 0.25 | 0.23-0.27 | 0.3 | 7 | 1 | 40 |
| Pelvis | Ischium ramus |  | 30 |  | 34 | 9 | 25 | 8 |  |  |  |  | 0.5 | 30 | 0.25 | 0.23-0.27 | 0.3 | 7 | 1 | 40 |
| Pelvis | Acetabulum | 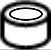 | 29 | 10 | 26 | 10 | 21 | 20 |  |  |  |  | 0.5  3.6 | 30  30 | 0.19 | 0.11-0.25 | 0.13 | 15 | 0.6 | 20 |
| Skull | Flat bones |  | 5.2 | 12 | 30 |  | 30 |  |  |  |  |  | 1.3  1.5 | 33  22 | 0.52 | 0.41-0.65 | 0.29 | 32 | 0.57 | 35 |
| Clavicle | Ends |  | 20 |  | 26 | 15 | 240 | 12 |  |  |  |  | 0.6 | 19 | 0.29 | 0.15-0.46 | 0.14 | 13 | 0.8 | 25 |
| Clavicle | Body acromial end |  | 56 | 7 | 26 | 15 | 24 | 12 | 12 | 5 | 12 | 8 | 1.8 | 2 | 0.13 | 0.08-0.18 | 0.19 | 31 | 0.8 | 25 |
| Clavicle | Body sternal end |  | 56 | 7 | 22 | 14 | 12 | 5 | 12 | 9 | 12 | 8 | 1.8 | 2 | 0.13 | 0.08-0.18 | 0.19 | 31 | 0.8 | 25 |
| Scapula | Glenoid |  | 16.9 | 8 | 30.7 | 11 | 22.3 | 18 |  |  |  |  | 0.9 | 28 | 0.22 | 0.09-0.47 | 0.24 | 42 | 0.96 | 23 |
| Scapula | Acromion |  | 8.8 | 18 | 32.4 | 4 | 25.2 | 4 |  |  |  |  | 0.8 | 13 | 0.22 | 0.09-0.47 | 0.24 | 42 | 0.96 | 23 |
| Scapula | Lateral margin |  | 30 |  | 3.5 | 3 | 10 | 12 |  |  |  |  | 0.8 | 13 | 0.22 | 0.09-0.47 | 0.24 | 42 | 0.96 | 23 |
| Sternum | Sternum body |  | 10 | 10 | 30 |  | 30 |  |  |  |  |  | 1.1 | 42 | 0.15 | 0.08-0.22 | 0.15 | 29 | 1.0 | 9 |
| Sternum | Sternum manubrium |  | 13 | 15 | 30 |  | 30 |  |  |  |  |  | 1.45 | 22 | 0.15 | 0.08-0.22 | 0.15 | 29 | 1.0 | 9 |
| C-vertebra | C-body 3-7 |  | 13 | 16 | 16 | 12 | 19 | 14 |  |  |  |  | 0.3 | 7 | 0.21 | 0.16-0.28 | 0.15 | 14 | 0.5 | 10 |
| C-vertebra | C-body 2 |  | 19.2 | 13 | 14.3 | 10 | 17.5 | 3 |  |  |  |  | 0.3 | 7 | 0.21 | 0.16-0.28 | 0.15 | 14 | 0.5 | 10 |
| C-vertebra | Cervical lateral 1 |  | 15 | 13 | 11.4 | 9 | 10.5 | 9 |  |  |  |  | 0.3 | 7 | 0.21 | 0.16-0.28 | 0.15 | 14 | 0.5 | 10 |
| T-vertebra | T- body |  | 27 | 7 | 28 | 11 | 33 | 9 |  |  |  |  | 1.3 | 6 | 0.21 | 0.16-0.28 | 0.15 | 14 | 0.5 | 10 |
| T-vertebra | T-transverse pr. |  | 12 | 9 | 18 | 11 | 10.6 | 13 |  |  |  |  | 1.3 | 16 | 0.16 | 0.11-0.28 | 0.15 | 15 | 0.6 | 15 |
| T-vertebra | T-spinous pr. |  | 10.3 | 15 | 50 | 4 | 5.1 | 20 |  |  |  |  | 1.3 | 16 | 0.16 | 0.11-0.28 | 0.15 | 15 | 0.6 | 15 |
| T-vertebra | T- lamina+inf. pr. |  | 32 | 12 | 10.2 | 14 | 4.2 | 13 |  |  |  |  | 1.3 | 16 | 0.16 | 0.11-0.28 | 0.15 | 15 | 0.6 | 15 |
| T-vertebra | T-superior pr. |  | 11.4 | 12 | 11.3 | 14 | 4.4 | 11 |  |  |  |  | 1.3 | 16 | 0.16 | 0.11-0.28 | 0.15 | 15 | 0.6 | 15 |
| L-vertebra | L- body |  | 27 | 12 | 35 | 8 | 47 | 9 |  |  |  |  | 1.3 | 16 | 0.16 | 0.11-0.28 | 0.15 | 15 | 0.6 | 15 |
| L-vertebra | L-transverse pr. |  | 12 | 8 | 23 | 9 | 8 | 13 |  |  |  |  | 0.4 | 50 | 0.15 | 0.1-0.19 | 0.1 | 13 | 0.6 | 16 |
| L-vertebra | L-spinous pr. |  | 24 | 13 | 31 | 6 | 6 | 17 |  |  |  |  | 0.4 | 50 | 0.15 | 0.1-0.19 | 0.1 | 13 | 0.6 | 16 |
| L-vertebra | L-lamina+inf.pr. |  | 20.4 | 10 | 12.7 | 13 | 4.1 | 17 |  |  |  |  | 1 | 34 | 0.15 | 0.1-0.19 | 0.1 | 13 | 0.6 | 16 |
| L-vertebra | L-superior pr. |  | 14 | 14 | 15 | 13 | 12 | 17 |  |  |  |  | 1 | 34 | 0.15 | 0.1-0.19 | 0.1 | 13 | 0.6 | 16 |

Standard relative uncertainties of DFs calculated for ^89^Sr and ^90^Sr were very similar. Therefore, Table S1.2 shows the uncertainties calculated for ^90^Sr only.

**Table S1.2. Summary of dose factor calculations for bone segments and skeleton sites of adult male.**

| Site | Segment | DF×10^-11^, Gy/s per Bq/g | | | | Standard relative uncertainty, % | | | | | |
| --- | --- | --- | --- | --- | --- | --- | --- | --- | --- | --- | --- |
|  |  |  |  |  |  | Inherent variability | | Introduced error | | Overall uncertainty | |
|  |  | ^90^Sr+^90^Y | | ^89^Sr | | *AM←TBV* | *AM←CBV* | *AM←TBV* | *AM←CBV* | *AM←TBV* | *AM←CBV* |
|  |  | *AM←TBV* | *AM←CBV* | *AM←TBV* | *AM←CBV* |  |  |  |  |  |  |
| Femur | Neck | 4.05 | 1.09 | 2.31 | 0.43 | 12 | 6 | 6 | 20 | 13 | 21 |
| Femur | Trochanter area | 2.69 | 0.87 | 1.55 | 0.32 | 14 | 16 | 6 | 21 | 15 | 26 |
| **Whole femur** | | **3.09** | **0.93** | **1.78** | **0.35** | **9** | **10** | **4.6** | **15** | **11** | **18** |
| Humeri | Proximal end | 1.64 | 0.82 | 0.90 | 0.40 | 51 | 19 | 1 | 17 | 51 | 25 |
| **Whole humeri** | | **1.64** | **0.82** | **0.90** | **0.40** | **51** | **19** | **1** | **17** | **51** | **25** |
| Ribs | 1, 2 | 2.56 | 2.70 | 1.50 | 1.32 | 28 | 21 | 1 | 12 | 28 | 25 |
| Ribs | 5-8 | 2.61 | 2.48 | 1.52 | 1.24 | 27 | 22 | 1 | 13 | 27 | 26 |
| Ribs | 3, 4, 9, 10 | 2.56 | 2.89 | 1.51 | 1.44 | 31 | 21 | 1 | 12 | 31 | 24 |
| Ribs | 11, 12 | 2.32 | 3.31 | 1.40 | 1.70 | 20 | 25 | 1 | 12 | 21 | 28 |
| **All ribs** | | **2.56** | **2.70** | **1.50** | **1.35** | **17** | **22** | **0.6** | **14** | **17** | **26** |
| Sacrum | Body 4-5 | 3.51 | 3.31 | 1.71 | 1.35 | 24 | 21 | 1 | 12 | 24 | 24 |
| Sacrum | Body 1 | 3.94 | 0.67 | 1.88 | 0.31 | 20 | 23 | 1 | 17 | 20 | 28 |
| Sacrum | Body 2-3 | 3.81 | 1.35 | 1.83 | 0.57 | 22 | 17 | 1 | 12 | 22 | 21 |
| Sacrum | Pedicle 1 | 3.54 | 2.06 | 1.75 | 0.91 | 26 | 16 | 1 | 12 | 26 | 20 |
| Sacrum | Pedicle 2 | 3.59 | 2.25 | 1.74 | 0.97 | 18 | 17 | 1 | 12 | 18 | 21 |
| Sacrum | Pedicle 3 | 3.41 | 2.56 | 1.70 | 1.11 | 23 | 19 | 1 | 12 | 23 | 23 |
| Sacrum | Pedicle 4 | 3.24 | 3.24 | 1.65 | 1.41 | 20 | 21 | 1 | 12 | 20 | 24 |
| Sacrum | Sacral ala 1 | 3.90 | 0.93 | 1.86 | 0.48 | 21 | 29 | 1 | 17 | 21 | 34 |
| Sacrum | Sacral ala 2 | 3.79 | 1.11 | 1.85 | 0.46 | 18 | 17 | 1 | 17 | 18 | 24 |
| Sacrum | Sacral ala 3-4 | 3.57 | 2.51 | 1.71 | 1.10 | 16 | 14 | 1 | 17 | 16 | 22 |
| **Whole sacrum** | | **3.79** | **1.33** | **1.83** | **0.59** | **9** | **8** | **0.4** | **5** | **9** | **10** |
| Pelvis | Iliac crest | 4.63 | 0.80 | 2.56 | 0.37 | 20 | 30 | 1 | 12 | 20 | 32 |
| Pelvis | Iliac ala | 4.31 | 1.78 | 2.44 | 0.82 | 21 | 67 | 1 | 12 | 21 | 68 |
| Pelvis | Iliac dorsal segment | 4.34 | 1.83 | 2.46 | 0.86 | 21 | 28 | 1 | 12 | 21 | 30 |
| Pelvis | Pubis ramus superior (lower) | 4.03 | 1.23 | 2.26 | 0.58 | 20 | 25 | 1 | 12 | 20 | 27 |
| Pelvis | Pubis ramus superior (upper) | 3.93 | 1.30 | 2.22 | 0.63 | 15 | 24 | 1 | 12 | 15 | 27 |
| Pelvis | Pubis ramus inferior | 5.58 | 1.04 | 3.12 | 0.54 | 23 | 33 | 1 | 12 | 23 | 35 |
| Pelvis | Ischium ramus | 5.82 | 0.73 | 3.20 | 0.37 | 6 | 25 | 1 | 17 | 6 | 30 |
| Pelvis | Acetabulum | 3.88 | 2.54 | 2.27 | 1.29 | 21 | 32 | 1 | 17 | 21 | 36 |
| **All pelvic bones** | | **4.66** | **1.31** | **2.61** | **0.63** | **7** | **17** | **0.4** | **5** | **7** | **18** |
| Skull | Flat bones | 9.64 | 3.95 | 5.68 | 1.72 | 21 | 55 | 15 | 16 | 26 | 57 |
| **Whole skull** | | **9.64** | **3.95** | **5.68** | **1.72** | **21** | **55** | **15** | **16** | **26** | **57** |
| Clavicle | Ends | 7.37 | 0.87 | 4.08 | 0.43 | 28 | 24 | 1 | 12 | 28 | 27 |
| Clavicle | Body acromial end | 2.98 | 2.13 | 1.78 | 0.89 | 19 | 24 | 13 | 16 | 23 | 28 |
| Clavicle | Body sternal end | 2.81 | 3.11 | 1.71 | 1.31 | 30 | 22 | 1 | 12 | 30 | 25 |
| **Whole clavicle** | | **5.00** | **1.72** | **2.84** | **0.75** | **20** | **14** | **2.8** | **8** | **20** | **17** |
| Scapula | Glenoid | 4.82 | 0.98 | 2.69 | 0.47 | 34 | 17 | 6 | 15 | 34 | 27 |
| Scapula | Acromion | 4.44 | 2.11 | 2.53 | 1.02 | 45 | 16 | 6 | 10 | 46 | 23 |
| Scapula | Lateral margin | 3.43 | 4.39 | 2.16 | 2.54 | 47 | 16 | 11 | 12 | 48 | 24 |
| **Whole scapula** | | **4.52** | **1.79** | **2.57** | **0.92** | **25** | **12** | **1** | **8** | **25** | **14** |
| Sternum | Sternum body | 3.48 | 1.80 | 1.97 | 0.81 | 23 | 18 | 1 | 12 | 23 | 22 |
| Sternum | Sternum manubrium | 3.57 | 1.57 | 2.00 | 0.66 | 23 | 25 | 1 | 12 | 23 | 28 |
| **Whole sternum** | | **3.51** | **1.71** | **1.98** | **0.75** | **17** | **15** | **0.7** | **9** | **17** | **17** |
| C-vertebra | C-body 3-7 | 4.89 | 0.65 | 2.77 | 0.35 | 14 | 20 | 1 | 12 | 14 | 24 |
| C-vertebra | C-body 2 | 4.96 | 0.40 | 2.79 | 0.22 | 16 | 15 | 1 | 12 | 16 | 20 |
| C-vertebra | Cervical lateral 1 | 4.81 | 3.39 | 2.75 | 1.49 | 14 | 19 | 5 | 12 | 15 | 22 |
| **All cervical vertebrae** | | **4.89** | **1.01** | **2.77** | **0.50** | **10** | **13** | **1.01** | **8** | **10** | **15** |
| T-vertebra | T- body | 4.25 | 0.51 | 2.34 | 0.27 | 22 | 20 | 1 | 17 | 22 | 26 |
| T-vertebra | T-transverse pr. | 2.84 | 3.14 | 1.65 | 1.45 | 22 | 18 | 1 | 12 | 22 | 21 |
| T-vertebra | T-spinous pr. | 2.23 | 7.14 | 1.40 | 3.56 | 30 | 28 | 7 | 13 | 31 | 31 |
| T-vertebra | T- lamina+inf. pr. | 2.89 | 6.55 | 1.79 | 3.51 | 31 | 37 | 9 | 13 | 32 | 39 |
| T-vertebra | T-superior pr. | 1.96 | 7.50 | 1.23 | 3.97 | 23 | 21 | 9 | 8 | 25 | 23 |
| **All thoracic vertebrae** | | **3.92** | **1.41** | **2.18** | **0.71** | **19** | **12** | **1** | **7** | **19** | **14** |
| L-vertebra | L- body | 3.93 | 0.38 | 1.89 | 0.21 | 21 | 25 | 1 | 17 | 21 | 30 |
| L-vertebra | L-transverse pr. | 3.25 | 1.75 | 1.89 | 0.95 | 18 | 19 | 1 | 12 | 18 | 23 |
| L-vertebra | L-spinous pr. | 2.89 | 1.86 | 1.68 | 1.00 | 24 | 41 | 1 | 12 | 24 | 42 |
| L-vertebra | L-lamina+inf.pr. | 2.03 | 5.37 | 1.26 | 2.90 | 20 | 30 | 7 | 13 | 21 | 33 |
| L-vertebra | L-superior pr. | 3.14 | 1.41 | 1.80 | 0.76 | 20 | 23 | 1 | 12 | 20 | 26 |
| **All lumbar vertebrae** | | **3.67** | **0.87** | **1.85** | **0.44** | **17** | **13** | **1** | **7** | **17** | **15** |
| **Whole skeleton** | | **4.20** | **1.54** | **2.33** | **0.74** | **25** | **36** | **6** | **11** | **26** | **38** |
